# Involvement of BCR::ABL1 in laminin adhesion of Philadelphia chromosome‐positive  acute lymphoblastic leukemia through upregulation of integrin α6

**DOI:** 10.1002/cnr2.2034

**Published:** 2024-04-05

**Authors:** Kazuya Takahashi, Thao Thu Thi Nguyen, Atsushi Watanabe, Hiroki Sato, Kinuko Saito, Minori Tamai, Daisuke Harama, Shin Kasai, Koshi Akahane, Kumiko Goi, Keiko Kagami, Masako Abe, Chiaki Komatsu, Yasuhiro Maeda, Kanji Sugita, Takeshi Inukai

**Affiliations:** ^1^ Department of Pediatrics, Faculty of Medicine University of Yamanashi Chuo Japan; ^2^ Department of Internal Medicine, Division of Hematology, Faculty of Medicine Kindai University Osakasayama Japan

**Keywords:** ALL, CD49f, imatinib, integrin α6, Philadelphia chromosome

## Abstract

**Background:**

Adhesion of cancer cells to extracellular matrix laminin through the integrin superfamily reportedly induces drug resistance. Heterodimers of integrin α6 (CD49f) with integrin β1 (CD29) or β4 (CD104) are major functional receptors for laminin. Higher CD49f expression is reportedly associated with a poorer response to induction therapy in childhood B‐cell precursor acute lymphoblastic leukemia (BCP‐ALL). Moreover, a xenograft mouse model transplanted with primary BCP‐ALL cells revealed that neutralized antibody against CD49f improved survival after chemotherapy.

**Aims:**

Considering the poor outcomes in Philadelphia chromosome (Ph)‐positive ALL treated with conventional chemotherapy without tyrosine kinase inhibitors, we sought to investigate an involvement of the laminin adhesion.

**Methods and results:**

Ph‐positive ALL cell lines expressed the highest levels of CD49f among the BCP‐ALL cell lines with representative translocations, while CD29 and CD104 were ubiquitously expressed in BCP‐ALL cell lines. The association of Ph‐positive ALL with high levels of CD49f gene expression was also confirmed in two databases of childhood ALL cohorts. Ph‐positive ALL cell lines attached to laminin and their laminin‐binding properties were disrupted by blocking antibodies against CD49f and CD29 but not CD104. The cell surface expression of CD49f, but not CD29 and CD104, was downregulated by imatinib treatment in Ph‐positive ALL cell lines, but not in their T315I‐acquired sublines. Consistently, the laminin‐binding properties were disrupted by the imatinib pre‐treatment in the Ph‐positive ALL cell line, but not in its T315I‐acquired subline.

**Conclusion:**

BCR::ABL1 plays an essential role in the laminin adhesion of Ph‐positive ALL cells through upregulation of CD49f.

## 1 INTRODUCTION

The bone marrow environment plays an essential role in normal hematopoiesis. In particular, the hematopoietic stem cell niche is an integral component of the extracellular matrix (ECM) in the bone marrow environment.^1^ Laminin, a major ECM component, is closely associated with normal hematopoiesis.^2^ Interaction of hematopoietic stem and progenitor cells with ECM laminins is mediated by the integrin superfamily of cell‐surface adhesion molecules as receptors for laminin.^3^ These interactions are reportedly involved in many cellular processes of hematopoietic stem cells, including cell cycle progression, differentiation, motility, and survival.^4^, ^5^ Among the integrin superfamily, heterodimers of integrin α6 (CD49f) chain assembling integrin β1 (CD29) or integrin β4 (CD104) chains are reportedly major functional receptors for ECM laminins in hematopoietic stem and progenitor cells.^2^, ^6^, ^7^, ^8^

In recent years, interactions between integrin receptors and ECM have also been noticed in diverse types of cancers, including B‐cell precursor acute lymphoblastic leukemia (BCP‐ALL).^9^ Indeed, integrin is involved in bone marrow homing,^10^ infiltration,^11^, ^12^ and localization^13^, ^14^ of BCP‐ALL cells. Moreover, decreased activity of integrin α4 (CD49d)‐CD29 heterodimer was reportedly associated with increased numbers of circulating lymphoblasts in BCP‐ALL patients.^15^ Of clinical importance, a higher *CD49d* (*ITGA4*) gene expression level at first relapse was reportedly associated with a poor prognosis in BCP‐ALL.^16^ Further, neutralized antibody against CD49d consistently sensitized primary ALLs to chemotherapy in a xenograft mouse model.^17^ In addition to CD49d, the clinical significance of CD49f in childhood BCP‐ALL has been highlighted by a recent comprehensive clinical study in which CD49f expression was associated with persistent minimal residual disease (MRD).^18^ Moreover, Gang et al.^19^ revealed that a higher *CD49f* (*ITGA6*) gene expression at diagnosis was associated with a poor response to induction therapy in childhood BCP‐ALL. The authors also demonstrated that neutralized antibody against CD49f improved survival after chemotherapy in xenograft mouse models transplanted with primary BCP‐ALL cells.^19^ Of clinical importance, CD49f was reportedly involved in central nervous system (CNS) infiltration of BCP‐ALL cells.^20^

The Philadelphia chromosome (Ph) is one of the common chromosomal abnormalities in BCP‐ALL. Although the prognosis of Ph‐positive ALL patients treated with standard chemotherapy alone was previously very poor, the development of tyrosine kinase inhibitors (TKIs) specific for BCR::ABL1 oncoprotein was a great innovation^21^, ^22^ that dramatically improved therapeutic outcomes in Ph‐positive ALL patients through the introduction of TKI‐combined chemotherapy.^23^, ^24^, ^25^ Considering the resistance of Ph‐positive ALL to standard chemotherapy, leukemia cell adhesion to ECM through integrin receptors might be involved in chemoresistance. Moreover, TKI treatment of Ph‐positive ALL may alter the expression of integrin receptors and subsequently leukemia cell adhesion to ECM.

In the present study, we investigated gene and cell surface expression of CD49f in BCP‐ALL cell lines and confirmed the highest expression levels in both Ph‐positive ALL cell lines and Ph‐positive ALL clinical samples. Indeed, we confirmed that Ph‐positive ALL cell lines attached to laminin through the CD49f‐CD29 heterodimer. Of note, imatinib treatment of the Ph‐positive ALL cell line downregulated CD49f expression and disrupted laminin adhesion, indicating that BCR::ABL1 itself is involved in high levels of CD49f expression and subsequently the laminin‐binding properties in Ph‐positive ALL.

## 2 MATERIALS AND METHODS

### 2.1 Cell lines

As listed in Table 1, 87 BCP‐ALL cell lines were analyzed, including 15 Ph‐positive cell lines, 2 Ph‐like cell lines, 14 *KMT2A* rearranged (*KMT2A*r) cell lines, 16 *TCF3::PBX1*‐positive cell lines, 4 *TCF3::HLF*‐positive cell lines, 6 *ETV6::RUNX1*‐positive cell lines, 19 *MEF2D*‐rearranged (*MEF2D*r) cell lines, and 11 other cell lines.^26^ KOPN, KOCL, YAMN, and YACL series of cell lines were sequentially established in our laboratory, as previously reported.^26^

**TABLE 1:** List of cell lines.

Translocations	(*n* = 87)	Cell lines
Ph‐positive	(*n* = 15)	KOPN30bi, KOPN55bi, KOPN56, KOPN57bi, KOPN66bi, KOPN72bi, YAMN73, KOPN83bi, YAMN91, TCCY, SU‐Ph2, NALM27, KCB1, SK9, Kasumi8
Ph‐like	(*n* = 2)	KOPN49, YCUB5
*KMT2A* rearranged	(*n* = 14)	KOPN1, KOPB26, KOCL33, KOCL44, KOCL45, KOCL50, KOCL51, KOCL58, KOCL69, KOCL77, YACL95, RS4;11, KOPN35, THP8
*TCF3::PBX1*‐positive	(*n* = 16)	KOPN‐K, KOPN34, KOPN36, KOPN54, KOPN60, KOPN63, YAMN90R, YAMN92, YCUB6, YCUB8, Kasumi2, THP‐4, SCMC‐L1, RCH697, PreALP
*TCF3::HLF*‐positive	(*n* = 4)	HALO1, YCUB2, Endokun, UOCB1
*ETV6::RUNX1*‐positive	(*n* = 6)	KOPN41, KOPN79, Reh, KOPN68, MBIT, KOPN87
*MEF2D*‐rearranged	(*n* = 19)	KOS20, KOPN39, KOPN61, KOPN62, KOPN70, KOPN71, YAMN74, YCUB4, YCUB7, P30/OHK, MBMY, THP5, THP7, L‐KUM, L‐ASK, YAMN96, Kasumi9, KOPN46, HBL3
B‐others	(*n* = 11)	KOPN84, Nalm6, KOPN75, KOPN40, KOPN32, KOPN85, SCMCL2, KCB4, KOPB38, KOPB59, YAMN93

YCUB and KCB series of cell lines were provided in 2014 (H. Goto) and sequentially established at Yokohama City University and Kanagawa Children's Medical Center. The THP series of cell lines, L‐KUM and L‐ASK were provided in 2014 (M. Minegishi) and sequentially established at Tohoku University. The MB series of cell lines were provided in 2014 (S. Iwamoto) and sequentially established at the Mie University Graduate School of Medicine. SU‐Ph2 was established at Kindai University and was provided in 2010. TCCY was provided in 2011 (Dr. Y. Sato) and established at the Tochigi Cancer Center. HALO1 and SK9 were provided in 1997 (Dr. T. Look of Dana‐Farber Cancer Institute, Boston, MA) and 2012 (Dr. S. Okabe) and established at Tokyo Medical University. Endokun was provided in 1997 (Dr. M. Endo) and established at Iwate Medical University. The Kasumi series of cell lines were provided in 2010 (Dr. T. Inaba) and sequentially established at Hiroshima University. SCMCL2 was provided in 2014 (Dr. J. Takita) and established at Saitama Children's Medical Center. P30/OHK and Nalm27 were purchased from ATCC in 2012. SU/SR was established from SU‐Ph2 after long‐term culture with increasing concentrations of imatinib and acquired the T315I mutation.^27^

KOPN55biR with the T315I mutation was established from KOPN55bi by homologous recombination using the CRISPR/Cas9 system.^28^ All cell lines were maintained in RPMI1640 medium supplemented with 10% fetal calf serum in a humidified atmosphere of 5% CO_2_ at 37°C.

### 2.2 Flow cytometric analysis

In brief, to block nonspecific binding, each cell line was resuspended in PBS after being washed twice with PBS, and incubated with human IgG antibody (#I2511, SIGMA) on ice for 15 min. After blocking, the cells (0.5 million cells/50 μL) were incubated on ice in the dark for 30 min with 5 μL/sample of phycoerythrin (PE)‐conjugated rat antihuman CD49f (#313612, BioLegend, 100 μg/mL), fluorescein isothiocyanate (FITC)‐conjugated mouse antihuman CD29 (#6603109, Beckman Coulter, 2 mg/mL), or PE‐conjugated mouse antihuman CD104 (#327808, BioLegend, 200 μg/mL). As isotype controls, the other aliquots of cells were incubated with PE‐conjugated rat IgG2b,κ (#553989, BD Pharmingen) or mouse IgG1 (#A07796, Beckman Coulter). After washing twice with ice‐cold PBS, the cells were analyzed using flow cytometry (FACSCalibur, BD Biosciences, San Jose, CA).

### 2.3 Real‐time RT‐PCR analysis

Total RNA was extracted from each cell line using TRIzol reagent (Thermo Fisher Scientific, Waltham, MA), as previously reported.^29^ Reverse transcription (RT) was performed using a random hexamer with Superscript IV reverse transcriptase (Thermo Fisher Scientific). For quantitative real‐time polymerase chain reaction (PCR) of *CD49f* (*ITGA6*), triplicated samples containing cDNA with TaqMan Universal PCR Master Mix and Gene Expression Product (Hs01041011_m1; Thermo Fisher Scientific) were amplified according to the manufacturer's protocol using Nalm6 as a control. As an internal control for relative gene expression, quantitative real‐time PCR for *ACTB* (#1808201, Thermo Fisher Scientific) was performed.

### 2.4 Laminin adhesion assay

For preparation of the laminin‐coated plate, flat‐bottom 96‐well microplates (Corning) were incubated with highly purified and refined laminin‐511 E8 fragments (Easy imatrix‐511, Matrixome Inc) at 0.5 μg/cm^2^. After overnight incubation at 4°C, the excessive laminin was aspirated. For the laminin‐uncoated plate, the plate was similarly incubated with PBS only. Following the instructions provided by the respective manufacturer, each cell line was preincubated with 25 μg/mL of anti‐CD49f (#313614, BioLegend),^30^ 12.5 μg/mL of anti‐CD29 (#921304, BioLegend),^31^ or 10 μg/mL of anti‐CD104 (#325‐820, Ancell) neutralizing antibodies or isotype control rat IgG2α,κ (#400502, BioLegend) (CD49f and CD29 blocking) or mouse IgG (#6602398, Beckman Coulter) (CD104 blocking) for 30 min. To verify the effect of imatinib on laminin adhesion, parental KOPN55bi cells or T315I‐acquired KOPN55biR cells^28^ were preincubated with 0.5 μM of imatinib for 7 days. After washing with PBS, the cells were resuspended in the medium at 0.5 × 10^6^/mL, and 100 μL of the cells was plated into each well of the laminin‐coated 96‐well plate in triplicate and allowed to attach to laminin for 3 h. After 3‐hour incubation of the cells on the plate with or without laminin coating, the plate was mechanically shaken using a plate mixer (Mikura Orbis Personal Single Plate Shaker, Mikura, West Sussex, UK) at 1300 rpm for 15 s. Then, the numbers of floating cells in the supernatant were evaluated after Trypan blue exclusion staining under an optical microscope. Meanwhile, the cell nuclei attached to the plate were fixed with 4% paraformaldehyde (Wako Pure Chemical Industries) for 20 min and stained by 1 μg/mL DAPI (#DM037, Dojindo Laboratories) for 20 min. In each cell nuclei photograph taken under a fluorescence microscope at 100‐fold magnification, three independent areas of 200 μm in diameter were evaluated by GIMP software (Free Software Foundation Inc.), and the numbers of attached cell nuclei were analyzed with ImageJ software (free software).

### 2.5 Statistical analysis and bioinformatic evaluation

All experiments were conducted in triplicate. Statistical significance was evaluated by the Mann–Whitney test or Student's *t*‐test for comparisons between groups. Gene expression levels in clinical samples of childhood BCP‐ALL patients were accessed from a database of the Nordic Society of Pediatric Hematology and Oncology (NOPHO) (GSE47051) and St. Jude Cloud PeCan (https://pecan.stjude.cloud/).

## 3 RESULTS

### 3.1 Ph‐positive ALL cells express higher levels of CD49f

**FIGURE 1:**
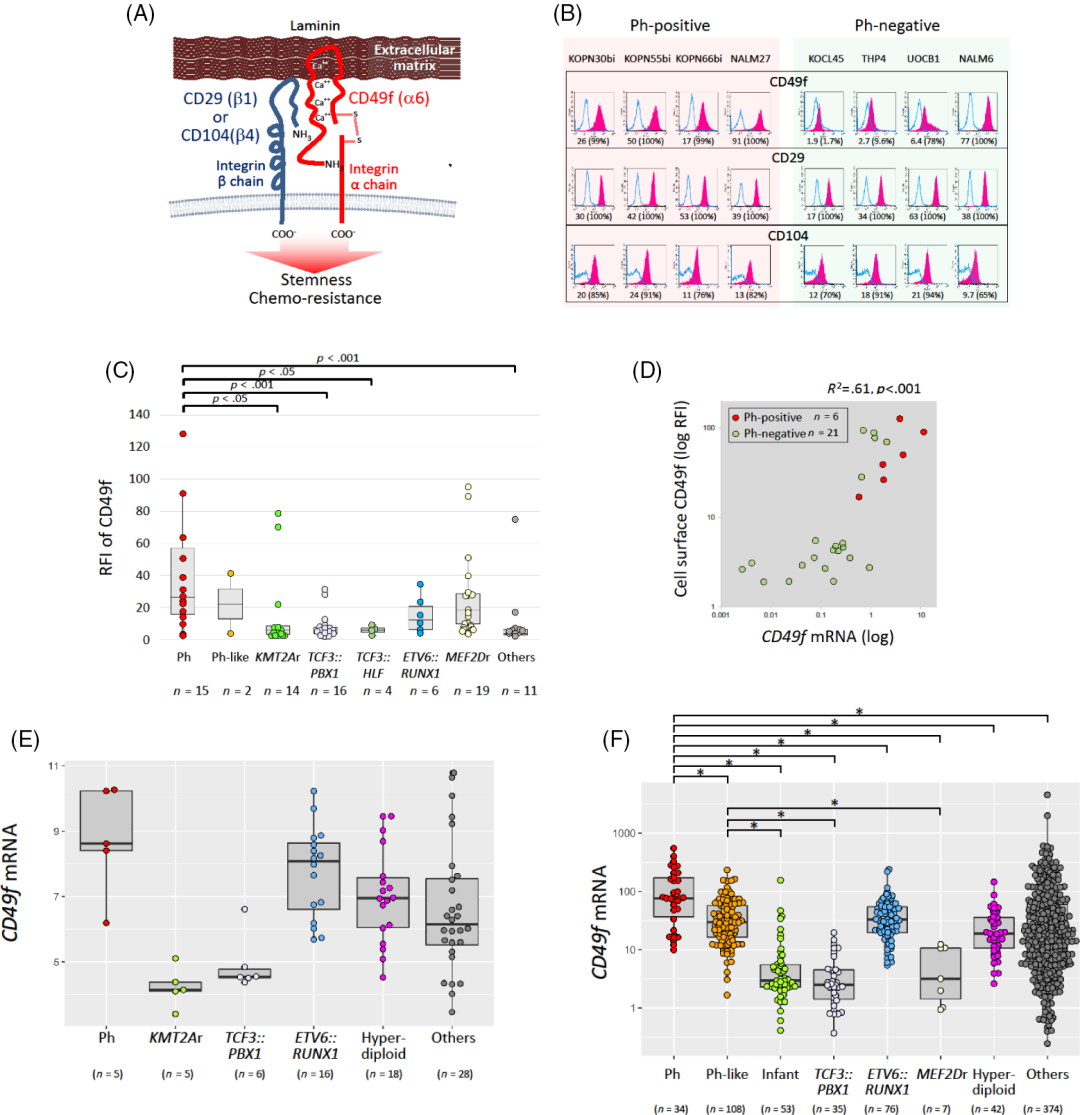
High CD49f expression level in Ph‐positive ALL. (A) Schematic representation of laminin binding of leukemia cells through the integrin α6 (CD49f) and β1 (CD29) or β4 (CD104) heterodimer. The illustration was created based on the illustration by Takeuchi that was published in a Japanese review article (*Jpn J Clin Immun*. 1994;17:515‐524). (B) Flow cytometric analyses of CD49f, CD29, and CD104 in four Ph‐positive (KOPN30bi, KOPN55bi, KOPN66bi, and NALM27) and four Ph‐negative (KOCL45, THP4, UOCB1, and NALM6) BCP‐ALL cell lines. Red‐filled peaks and blue lines indicate histograms of the specific antibody and isotype control, respectively. The relative fluorescence intensity (RFI) and % positivity of each cell line are indicated below. (C) Comparison of CD49f expression levels (RFI) among 7 representative translocations in 87 BCP‐ALL cell lines. The *p*‐value in the Mann–Whitney analysis is indicated at the top when significant. (D) Correlation between gene (horizontal axis) and cell surface expression (RFI; vertical axis) levels of CD49f in 27 BCP‐ALL cell lines, including 6 Ph‐positive ALL cell lines (red circles). The correlation coefficient is indicated at the top. (E, F) Association of representative chromosomal abnormalities with *CD49f* gene expression levels in childhood BCP‐ALL samples in the NOPHO (E) and St. Jude (F) cohorts. Asterisks are shown when the *p* values in the Kruskal–Wallis test are <.01 with Ph‐positive ALL samples or Ph‐like ALL samples.

CD49f binds to laminin as a heterodimer in combination with CD29 or CD104 (Figure 1A).^32^ First, we analyzed the cell surface expression levels of CD49f, CD29, and CD104 in eight representative BCP‐ALL cell lines, including four Ph‐positive cell lines, using flow cytometry (Figure 1B). Of note, all four Ph‐positive cell lines expressed CD49f at a high level, while only one of the four Ph‐negative cell lines expressed CD49f at a high level. In contrast to CD49f, CD29, and CD104 were almost ubiquitously expressed in both Ph‐positive and Ph‐negative cell lines. These observations suggested the possibility that the expression level of CD49f may crucially be involved in the laminin‐binding ability of BCP‐ALL cells. Thus, we further investigated CD49f expression in 87 BCP‐ALL cell lines, and a relatively high level (positive rate >50%) of expression was observed in the majority of BCP‐ALL cell lines (58 of 87 cell lines) (Figure S1). We then investigated the association of cell surface expression levels of CD49f with cytogenetic abnormalities.

Among the six representative translocations, Ph‐positive BCP‐ALL cell lines showed the highest cell surface expression level of CD49f. The relative fluorescence intensity (RFI) of CD49f in 15 Ph‐positive cell lines was significantly higher than that in 14 *KMT2A*‐rearranged cell lines, 16 *TCF3::PBX1*‐positive cell lines, and 4 *TCF3::HLF*‐positive cell lines (Figure 1C). Moreover, although only two cell lines were available, the RFIs in the two Ph‐like ALL cell lines were almost similar to those in Ph + ALL cell lines (Figure 1C). When the percentage of positive cells was compared (Figure S1), the CD49f expression level in Ph‐positive cell lines was also significantly higher than that in *TCF3::PBX1*‐positive cell lines. We next evaluated the correlation between the gene and cell surface expression levels of CD49f in 27 representative BCP‐ALL cell lines by RT‐PCR analysis. A clear positive correlation (*R* ^2^ = .61) between the gene and cell surface expression levels of CD49f was confirmed (Figure 1D; Figure S2).

Since a significant positive correlation between gene and cell surface expression levels of CD49f was confirmed in BCP‐ALL cell lines, we next evaluated the association of *CD49f* gene expression levels with cytogenetic abnormalities in childhood BCP‐ALL samples using two public databases. In the database of the NOPHO study, Ph‐positive ALL samples showed the highest level of *CD49f* mRNA expression among the samples, including *KMT2A*‐rearranged and *TCF3::PBX1*‐positive ALL (Figure 1E), although no statistical significance was observed probably due to a relatively limited number of samples. In the database of St Jude Children's Research Hospital, Ph‐positive ALL samples showed the highest level of *CD49f* mRNA expression: the *CD49f* gene expression level of Ph‐positive ALL samples was significantly higher than that of any other subgroups including infant ALL samples and *TCF3::PBX1*‐positive ALL samples (Figure 1F). Moreover, Ph‐like ALL samples also showed relatively higher levels of *CD49f* gene expression than infant ALL samples, *TCF3::PBX1*‐positive ALL samples, and *MEF2D*‐rearranged ALL samples.

Next, we evaluated the gene expression levels of *CD29* and *CD104* in the two databases. In comparison with the *CD49f* gene expression level, the gene expression levels of *CD29* and *CD104* were relatively ubiquitous in the ALL samples in both databases (Figure S3A–D). These observations in both cell lines and clinical databases indicated that Ph‐positive ALL is associated with high gene and cell surface expression levels of CD49f.

### 3.2 Ph‐positive ALL cell lines adhere to laminin through CD49f‐CD29 heterodimer

**FIGURE 2:**
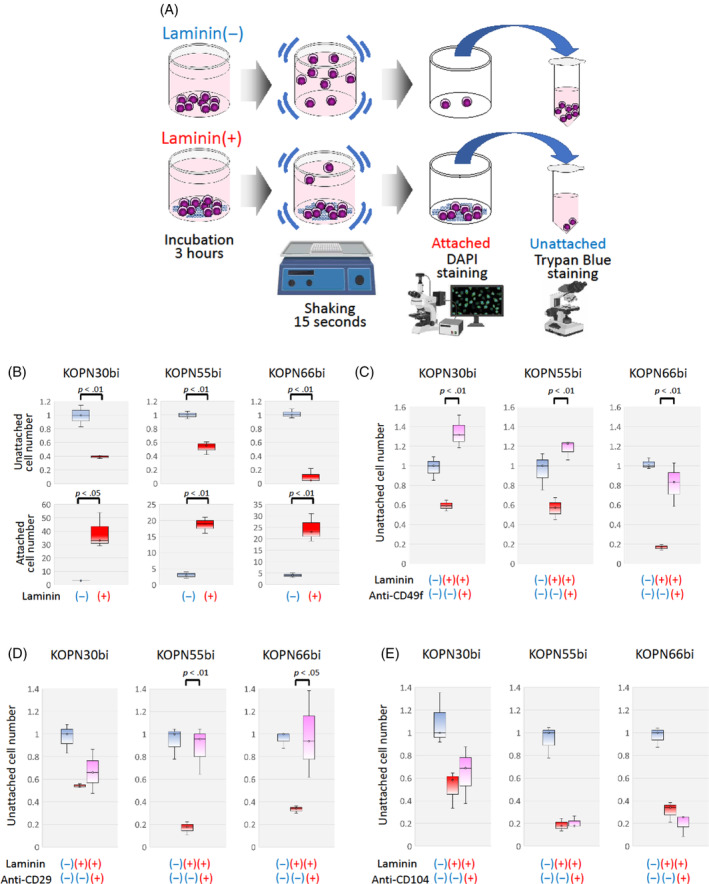
Laminin‐adhesion of Ph‐positive ALL cell lines through the CD49f‐CD29 heterodimer. (A) Experimental workflow of the laminin‐binding assay. The illustration was created with BioRender.com. (B) Comparison of numbers of unattached cells (top panel) and attached cell nuclei (bottom panel) between laminin‐uncoated (−) and laminin‐coated (+) plates. In the top panel, the vertical axis indicates a relative unattached cell number in the laminin‐coated plate to that in the laminin‐uncoated plate. In the bottom panel, the vertical axis indicates number of attached cell nuclei. (C) Effect of anti‐CD49f blocking antibody or isotype IgG on the numbers of unattached cells in the laminin‐coated plates. (D) Effect of anti‐CD29 blocking antibody or isotype IgG on the numbers of unattached cells in the laminin‐coated plates. (E) Effect of anti‐CD104 blocking antibody or isotype IgG on the numbers of unattached cells in the laminin‐coated plates. The vertical axis indicates a relative unattached cell number to that in the laminin‐uncoated plate in the absence of specific antibody. The *p*‐value in the paired *t*‐test is indicated at the top when significant.

Since Ph‐positive ALL cell lines expressed CD49f, CD29, and CD104 at relatively high levels, Ph‐positive ALL cells may attach to laminin through heterodimers between CD49f and CD29 or CD104. To verify this possibility, we analyzed adhesion to laminin using three Ph‐positive ALL cell lines (KOPN30bi, KOPN55bi, and KOPN66bi). These three cell lines expressed CD49f, CD29, and CD104 on their cell surfaces at high levels (Figure 1B) and showed floating phenotypes in a standard liquid culture system. In contrast, NALM27 could not be used in the experiment since it nonspecifically attached to the culture plate without laminin‐coating in a standard liquid culture system. After 3‐hour incubation of the cells in the plates coated with or without laminin, the plates were mechanically shaken using a plate mixer for 15 s (Figure 2A). Subsequently, we separately harvested floating cells in the culture medium, allowed the floating cells to attach to the culture plate, and evaluated the numbers of unattached cells and attached cell nuclei in triplicate using Trypan blue staining and DAPI staining, respectively.

In all three Ph‐positive ALL cell lines, the number of unattached cells dramatically decreased in the laminin‐coated plate in comparison with those in the laminin‐uncoated plate (Figure 2B). On the other hand, the number of attached cell nuclei stained with DAPI in the laminin‐coated plate was significantly higher than that in the laminin‐uncoated plate (Figure 2B; Figure S4). These observations clearly demonstrated that these three Ph‐positive ALL cell lines adhered to laminin.

Then, to directly investigate the involvement of a heterodimer between CD49f and CD29 and/or CD104, we evaluated the effects of blocking antibodies specific for CD49f, CD29, or CD104 on the cell adhesion of the three Ph‐positive ALL cell lines to laminin. Each cell line was preincubated with blocking antibodies or isotype IgG for 30 min and then incubated in the laminin‐coated plates for 3 h (Figure S5). After 15 s of mechanical shaking using a plate mixer, the floating cells and the attached cells were separately harvested for cell counts. Upon pretreatment with anti‐CD49f antibody, the numbers of unattached cells in all three Ph‐positive ALL cell lines were significantly increased in comparison with those that had been pretreated with isotype IgG (Figure 2C).

Similar to the anti‐CD49f antibody, upon pretreatment with anti‐CD29 antibody, the numbers of unattached cells in two of the three Ph‐positive ALL cell lines were significantly increased in comparison with those that had been pretreated with isotype IgG (Figure 2D). In contrast, upon pretreatment with anti‐CD104 antibody, the numbers of unattached cells in all of the three Ph‐positive ALL cell lines were almost unchanged in comparison with those that had been pretreated with isotype IgG (Figure 2E). Completely reciprocal patterns were observed in the number of attached cell nuclei to laminin‐coated plates (Figure S6A–F): the numbers of attached cell nuclei were significantly decreased upon blocking with anti‐CD49f and anti‐CD29 antibodies in all three Ph‐positive ALL cell lines, while the numbers were nearly unchanged upon blocking with anti‐CD104 antibody. These observations clearly demonstrated that Ph‐positive ALL cell lines bind to laminin mainly through heterodimers composed of CD49f and CD29.

### 3.3 BCR::ABL1 kinase activity induces a high level of CD49f expression and laminin‐binding in Ph‐positive ALL cell lines

**FIGURE 3:**
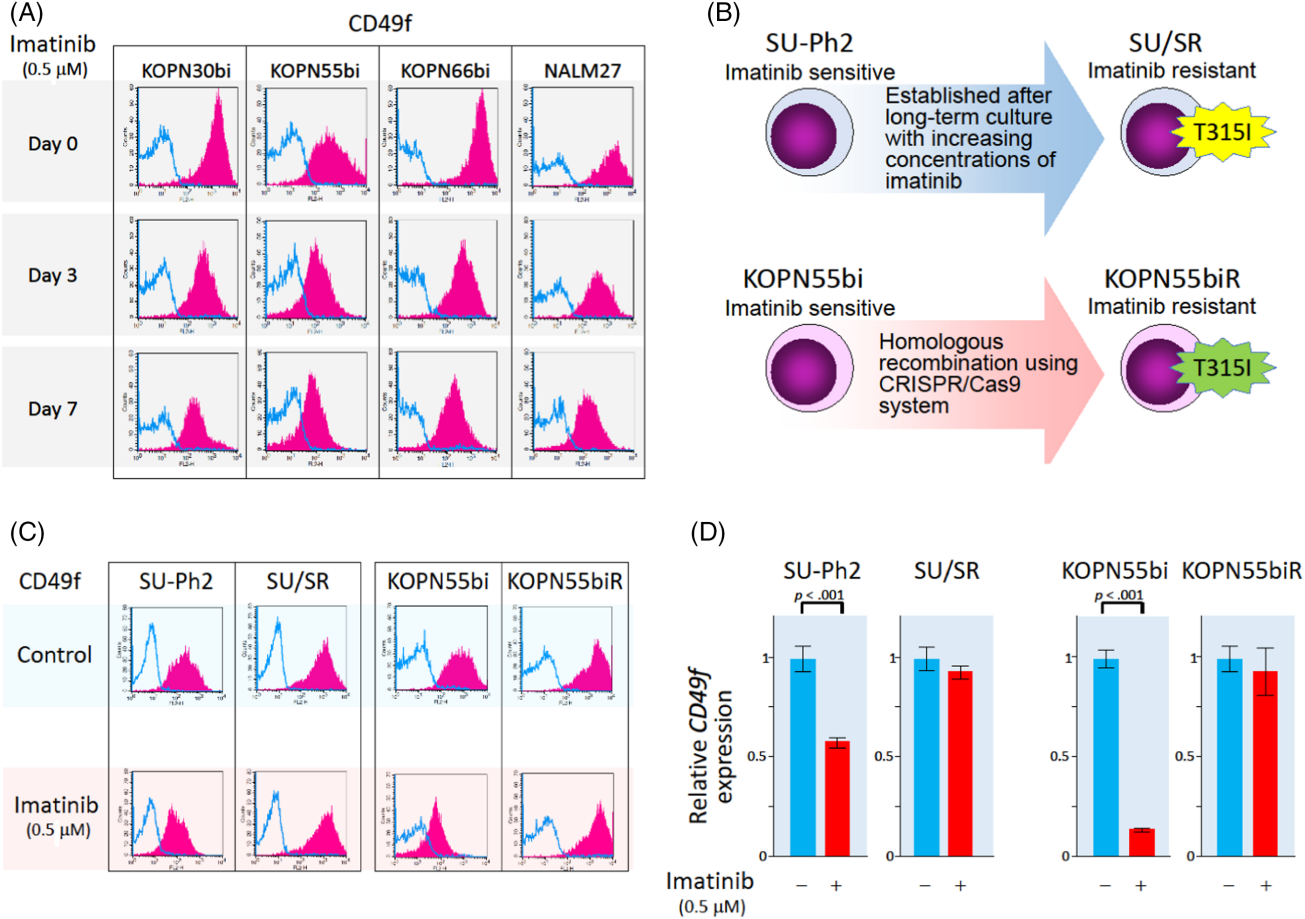
Imatinib treatment downregulates CD49f expression in Ph‐positive ALL cell lines. (A) Flow cytometric analysis of CD49f expression in four Ph‐positive ALL cell lines after 0 (top panel), 3 (middle panel), and 7 (bottom panel) days of incubation with 0.5 μM of imatinib. Red‐filled peaks and blue lines indicate histograms of the specific antibody and isotype control, respectively. (B) Schematic representation of two pairs of imatinib‐sensitive parental cell lines (SU‐Ph2 and KOPN55bi) and their T315I‐acquired sublines (SU/SR and KOPN55biR). (C) Flow cytometric analysis of CD49f expression in two pairs of imatinib‐sensitive parental cell lines and their T315I‐acquired sublines after imatinib (0.5 μM for 3 days) treatment. Red‐filled peaks and blue lines indicate histograms of the specific antibody and isotype control, respectively. (D) Gene expression level of *CD49f* in two pairs of imatinib‐sensitive parental cell lines and their T315I‐acquired sublines after imatinib treatment (0.5 μM for 24 h). The *p*‐value in the paired *t*‐test is indicated at the top when significant.

Regarding the underlying mechanism for higher CD49f expression in Ph‐positive ALL cells, we hypothesized that BCR::ABL1 itself may upregulate gene and cell surface expression levels of CD49f. To verify this possibility, we investigated the CD49f expression level of Ph‐positive ALL cell lines after treatment with imatinib. First, we treated four Ph‐positive ALL cell lines (KOPN30bi, KOPN55bi, KOPN66bi, and NALM27) with 0.5 μM of imatinib and evaluated their cell surface expression levels of CD49f, CD29, and CD104 by flow cytometry. When these four Ph‐positive ALL cell lines were treated with 0.5 μM of imatinib, BCR::ABL1 protein was dephosphorylated after 12‐hour treatment (Figure S7). Upon 3‐day treatment with imatinib, moderate numbers of cells were in the G0/G1 phase without inducing significant apoptosis (Figure S8). As expected, imatinib treatment gradually reduced cell surface expression of CD49f in all four Ph‐positive ALL cell lines (Figure 3A; Figure S9A,B). In contrast, imatinib treatment did not significantly change the cell surface expression levels of CD29 and CD104 in all four Ph‐positive leukemia cell lines (Figure S10A,B).

To more directly investigate the involvement of BCR::ABL1 in higher CD49f expression levels in Ph‐positive leukemia cells, we analyzed two pairs of Ph‐positive ALL cell lines, that is, the parental lines and their T315I acquired imatinib‐resistant sublines. In the SU‐Ph2 and SU/SR pair,^27^ SU/SR was established from SU‐Ph2 after long‐term culture with increasing concentrations of imatinib. SU/SR acquired the imatinib‐resistant phenotype as a result of T315I mutation (Figure 3B). In the KOPN55bi and KOPN55biR pair,^28^ KOPN55biR with T315I mutation was established from KOPN55bi by homologous recombination using the CRISPR/Cas9 system (Figure 3B). When treated with imatinib, consistent with their phosphorylation status of BCR::ABL1 protein (Figure S11), the cell surface expression levels of CD49f were downregulated in the parental SU‐Ph2 and KOPN55bi but not in the T315I‐acquired SU/SR and KOPN55biR (Figure 3C; Figure S12A,B). The identical pattern was observed in real‐time RT‐PCR analysis of *CD49f* gene expression (Figure 3D): the *CD49f* gene expression level was significantly reduced by imatinib treatment in the parental SU‐Ph2 and KOPN55bi, while it was nearly unchanged by imatinib treatment in the T315I‐acquired SU/SR and KOPN55biR. In contrast to CD49f, the cell surface expression levels of CD29 and CD104 were nearly unchanged by imatinib treatment in both pairs of Ph‐positive leukemia cell lines (Figure S13A,B).

**FIGURE 4:**
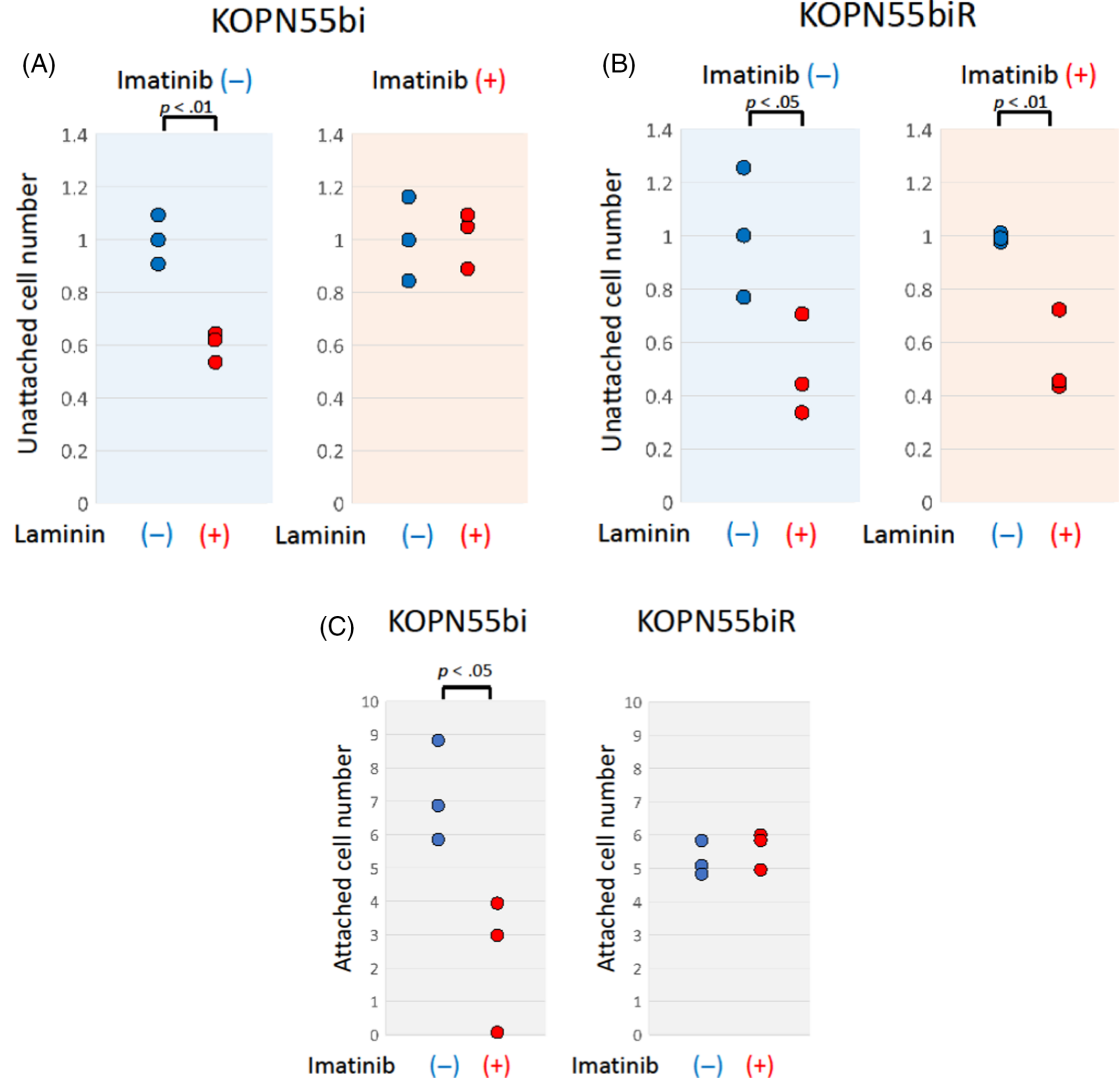
Imatinib treatment disrupts laminin binding in a Ph‐positive ALL cell line. (A, B) Comparison of unattached cell numbers of imatinib‐untreated (left panel) and imatinib‐pretreated (0.5 μM for 7 days; right panel) KOPN55bi (A) and KOPN55biR (B) cells in the laminin‐uncoated (−) and laminin‐coated (+) plates. The vertical axis indicates a relative unattached cell number in the laminin‐coated plate to that in the laminin‐uncoated plate. (C) Comparison of attached cell numbers of imatinib‐untreated and imatinib‐pretreated (0.5 μM for 7 days) KOPN55bi (left panel) and KOPN55biR (right panel) cells in the laminin‐coated plates. The vertical axis indicates number of attached cell nuclei. The *p*‐value in the paired *t*‐test is indicated at the top when significant.

Considering that heterodimers composed of CD49f and CD29 mainly mediated the laminin‐binding properties of Ph‐positive ALL cell lines, downregulation of CD49f by imatinib treatment may disrupt the laminin adhesion of these cell lines. SU‐Ph2 and SU/SR could not be used in the laminin adhesion assay, since SU‐Ph2 and SU/SR nonspecifically attached to the culture plate in the standard liquid culture system. In contrast, KOPN55bi and KOPN55biR showed a floating phenotype in the standard liquid culture system, and, thus, we performed the laminin adhesion assay using the KOPN55bi and KOPN55biR pair. We evaluated the laminin‐binding properties of the KOPN55bi and KOPN55biR pair after 7‐day pretreatment with 0.5 μM of imatinib. In the parental KOPN55bi cells cultured in the absence of imatinib, as already observed (Figure 2B), the number of unattached cells in the laminin‐coated plate was significantly lower than that in the laminin‐uncoated plate (Figure 4A). Of note, in the parental KOPN55bi cells pretreated with imatinib, the number of unattached cells in the laminin‐coated plate was unchanged from that in the laminin‐uncoated plate (Figure 4A). In contrast, in the T315I‐acquired KOPN55biR cells, even after imatinib pretreatment, the number of unattached cells in the laminin‐coated plate was significantly decreased in comparison with that in the laminin‐uncoated plate (Figure 4B). Consistently, the number of attached cell nuclei to the laminin‐coated plate was significantly decreased by imatinib pretreatment in the parental KOPN55bi cells, but was unchanged by imatinib pretreatment in the T315I‐acquired KOPN55biR cells (Figure 4C; Figure S14). These observations demonstrated that imatinib pretreatment disrupted the laminin‐binding properties of KOPN55bi through downregulation of CD49f.

## 4 DISCUSSION

In the present study, using a large panel of BCP‐ALL cell lines, we confirmed that Ph‐positive ALL cell lines had the highest cell surface and gene expression levels of CD49f. Moreover, the cell surface expression level of CD49f in BCP‐ALL cell lines was highly correlated with the gene expression level of *CD49f*. We also confirmed higher *CD49f* gene expression levels in Ph‐positive ALL samples using two databases of childhood BCP‐ALL cohorts. Of note, a recent study in patients with childhood BCP‐ALL treated with the NOPHO protocol revealed a significant association of CD49f expression with persistent MRD.^18^ Moreover, a recent report by Gang et al.^19^ revealed that a higher *CD49f* gene expression level at diagnosis was associated with poor response to induction therapy in childhood high‐risk BCP‐ALL patients (excluding Ph‐positive patients): the *CD49f* gene expression level in leukemia cells at diagnosis was significantly higher in the high‐risk group. In this context, it has been reported that high level of MRD was detectable at the end of induction therapy in the majority of childhood Ph‐positive ALL patients who were treated with conventional induction chemotherapy.^33^ Thus, higher CD49f expression in Ph‐positive ALL might be partially associated with poor clinical response to conventional induction therapy.

**FIGURE 5:**
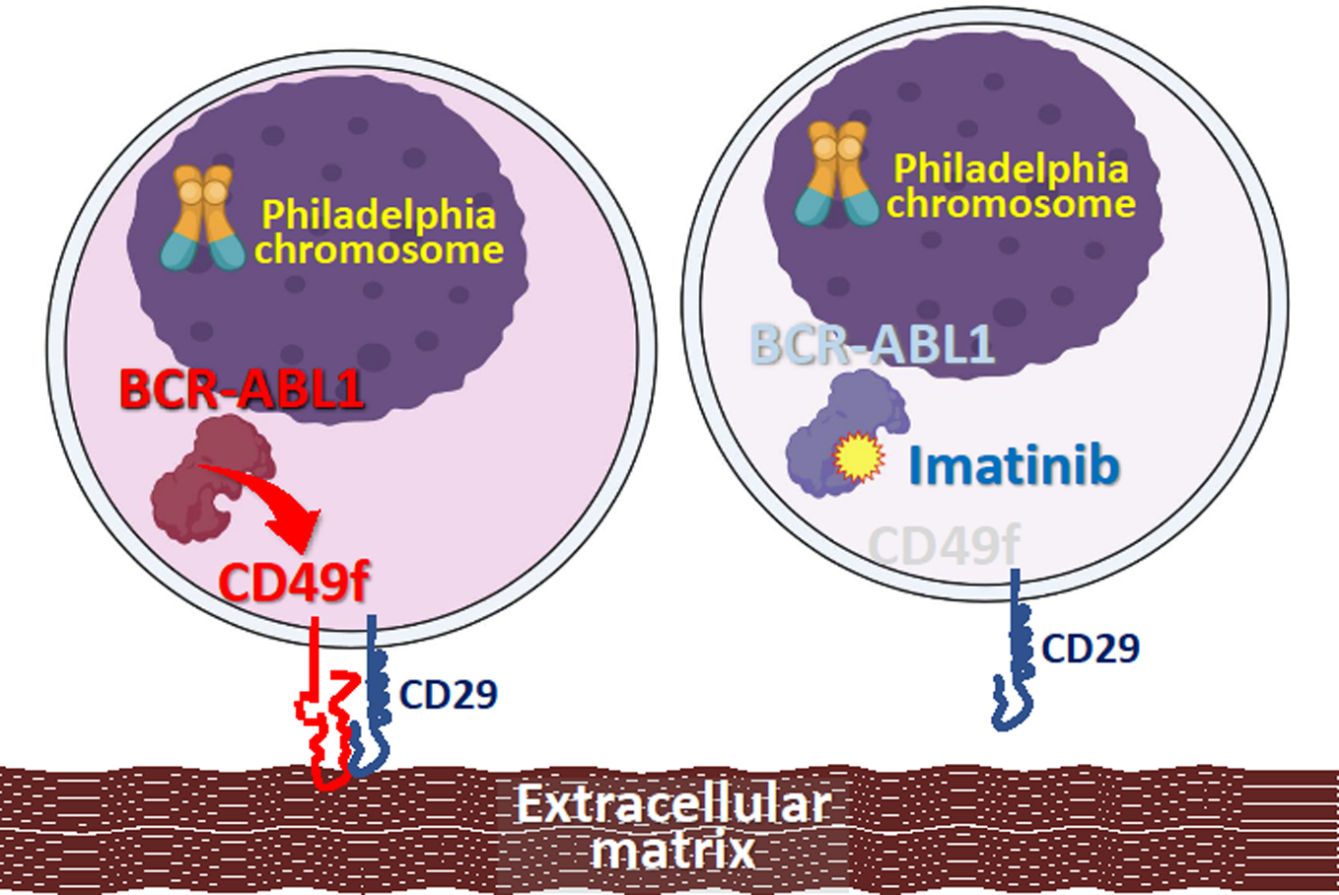
Imatinib treatment disrupts the ECM‐binding properties of Ph‐positive ALL cells through downregulation of the laminin receptor component CD49f. The illustration was created with BioRender.com.

In the present study, CD29 and CD104, two *β* subunits of CD49f‐associated heterodimer,^34^ were ubiquitously expressed in BCP‐ALL cell lines, regardless of their Ph‐positivity. Moreover, we confirmed that the laminin‐binding properties in three Ph‐positive BCP‐ALL cell lines were markedly disrupted by preincubation with blocking antibodies against CD49f and CD29, but not CD104. These observations collectively indicated that Ph‐positive BCP‐ALL cell lines attached to laminin mainly through a heterodimer composed of CD49f and CD29. Of clinical importance, it has been recently reported that adhesion of leukemia cells to an extracellular matrix, such as laminin, induces drug resistance.^35^, ^36^ Under these circumstances, although not directly confirmed in the present study, laminin adhesion of Ph‐positive ALL cells through the heterodimer composed of CD49f and CD29 may be one of the underlying mechanisms responsible for the poor clinical response to conventional induction therapy (Figure 5).

Interestingly, imatinib treatment of four Ph‐positive ALL cell lines downregulated CD49f expression, but not CD29 expression. Of note, in two pairs of parental TKI‐sensitive Ph‐positive ALL cell lines and their T315I‐acquired sublines, imatinib treatment downregulated CD49f expression in the parental cell lines but not in their T315I‐acquired sublines. Moreover, in one pair of the cell line with the floating feature, imatinib pretreatment disrupted laminin adhesion in the parental cell line but not in the T315I‐acquired subline. These observations indicated that the tyrosine kinase activity of BCR::ABL1 itself is involved in increasing the level of CD49f expression and, subsequently, the laminin‐binding properties in Ph‐positive ALL (Figure 5).

CD49f is considered to be one of the robust biomarkers for flow cytometric evaluation of MRD due to high levels of cell surface expression in BCP‐ALL.^37^, ^38^ Indeed, our observations supported the utility of CD49f as a biomarker to specifically detect Ph‐positive ALL cells. However, since we found that imatinib treatment downregulates CD49f in Ph‐positive ALL cell lines, application of CD49f to the flow cytometric MRD evaluation in Ph‐positive ALL patients treated with imatinib‐combined chemotherapy must be considered cautiously.

There are several limitations in this study. First, this was an in vitro study using leukemic cell lines, although our findings seemed to be consistent with the previous findings in xenograft models of clinical samples. Second, the laminin adhesion properties of Ph‐positive leukemia cell lines were not fully elucidated in this study since several cell lines (NALM27, SU‐Ph2, and SU/SR) became attached to the culture plate without laminin‐coating. Third, the possible involvement of other integrin members, such as integrin α4 (CD49d) was not evaluated in the present study.

Recently, it was reported that CD49f plays an essential role in the CNS invasion of ALL cells^20^: ALL blasts in cranial bone marrow attach to the outer wall of bridging vessels through CD49f‐laminin interaction and subsequently migrate into a subdural lesion in the CNS. In this scenario, a higher level of CD49f expression might be associated with a higher incidence of CNS invasion in ALL patients. Of note, a high incidence of meningeal leukemia was reported in the lymphoid blast crisis of CML patients.^39^ Moreover, high incidence of CNS leukemia at diagnosis was reported in childhood Ph‐positive ALL^40^ and a trend toward higher frequency of initial CNS involvement was reported in adult Ph‐positive ALL.^41^ Since we confirmed that laminin binding of Ph‐positive ALL cell lines is mediated through the heterodimer composed of CD49f and CD29, higher levels of CD49f expression in Ph‐positive ALL might, at least partly, be involved in higher incidence of CNS invasion. In this context, we confirmed that imatinib treatment disrupted the laminin‐binding properties of the Ph‐positive ALL cell line as a result of CD49f downregulation. Although penetration of imatinib into the CNS fluid was reported to be poor,^42^ imatinib treatment may reduce the risk of further CNS invasion of residual leukemia cells by downregulating CD49f expression in Ph‐positive ALL patients.

In Ph‐positive ALL, a deeper level of remission was reportedly achieved at the end of induction therapy in the patients treated with TKI combined induction chemotherapy compared with those treated with conventional induction therapy without TKI.^33^ Moreover, combinations of TKIs with conventional chemotherapy dramatically improved the therapeutic outcome in both adult^21^, ^43^, ^44^, ^45^ and childhood^46^ Ph‐positive ALL patients. Of note, in the NOG mice inoculated with the primary Ph‐negative BCP‐ALL sample,^19^ administration of anti‐CD49f blocking antibody significantly improved survival after chemotherapy consisting of vincristine, dexamethasone, and asparaginase.

This finding in the in vivo mice model suggested that ECM binding of BCP‐ALL cells through the laminin/CD49f interaction might be involved in the poor response of BCP‐ALL to induction therapy. Moreover, in the mice p210 *BCR::ABL1*‐transduced ALL model,^19^ in vivo conditional knockout of the integrin α6 gene sensitized the leukemia cells to nilotinib treatment, suggesting that the laminin/CD49f interaction might also be involved in the TKI‐resistance of Ph‐positive ALL. In the present study, we showed that imatinib treatment effectively disrupted the laminin‐binding properties of Ph‐positive ALL cell lines through downregulation of CD49f.

Considering these findings with our previous findings, attenuated ECM binding of Ph‐positive ALL cells through the diminished laminin/CD49f interaction by TKI treatment might be one of the underlying mechanisms of TKI‐combined chemotherapy.

## AUTHOR CONTRIBUTIONS

**Kazuya Takahashi:** Formal analysis (lead); investigation (lead); visualization (equal); writing ‐ original draft (equal); writing ‐ review & editing (equal). **Thao Thu Thi Nguyen:** Formal analysis (equal); investigation (equal); validation (equal). **Atsushi Watanabe:** Formal analysis (equal); investigation (equal); validation (equal). **Hiroki Sato:** Investigation (equal). **Kinuko Saito:** Investigation (equal). **Minori Tamai:** Investigation (equal). **Daisuke Harama:** Investigation (equal). **Shin Kasai:** Investigation (equal). **Koshi Akahane:** Investigation (equal). **Kumiko Goi:** Funding acquisition (equal); investigation (equal). **Keiko Kagami:** Formal analysis (equal); investigation (equal); methodology (equal). **Masako Abe:** Formal analysis (equal); investigation (equal); methodology (equal). **Chiaki Komatsu:** Formal analysis (equal); investigation (equal); methodology (equal). **Yasuhiro Maeda:** Resources (equal). **Kanji Sugita:** Funding acquisition (equal); supervision (equal). **Takeshi Inukai:** Conceptualization (lead); formal analysis (lead); funding acquisition (equal); investigation (lead); methodology (equal); project administration (lead); supervision (equal); visualization (equal); writing ‐ original draft (equal); writing ‐ review & editing (equal).

## CONFLICT OF INTEREST STATEMENT

The authors have stated explicitly that there are no conflicts of interest in connection with this article.

## ETHICS STATEMENT

Approval of the research protocol by an Institutional Reviewer Board: N/A.

## Supporting information


**Figure S1.** High CD49f expression levels in Ph‐positive ALL. Comparison of cell surface CD49f expression (%; vertical axis) levels in Ph‐positive ALL cell lines with those in each of 6 representative translocations among 87 BCP‐ALL cell lines. The *p*‐value in the Mann–Whitney analysis is indicated at the top when significant.
**Figure S2.** Correlation between gene and cell surface expression levels of CD49f. Correlation between gene (horizontal axis) and cell surface expression (%; vertical axis) levels of CD49f in 27 BCP‐ALL cell lines, including 6 Ph‐positive ALL cell lines (red circles). The correlation coefficient is indicated at the top.
**Figure S3.** *CD29* and *CD104* gene expression levels in Ph‐positive ALL samples. (A and B) Association of representative chromosomal abnormalities with *CD29* gene expression (vertical axis) levels in childhood BCP‐ALL samples of the NOPHO (A) and St. Jude (B) cohorts. (C and D) Association of representative chromosomal abnormalities with *CD104* gene expression (vertical axis) levels in childhood BCP‐ALL samples of the NOPHO (C) and St. Jude (D) cohorts.
**Figure S4.** Laminin adhesion of Ph‐positive ALL cell lines. Images of three Ph‐positive ALL cell lines (KOPN30bi, KOPN55bi, KOPN66bi) attached to laminin‐uncoated (−) (upper panel) or laminin‐coated (+) (lower panel) plates after DAPI staining.
**Figure S5.** Experimental workflow of blocking assay of laminin adhesion by specific antibodies.
**Figure S6.** Laminin‐adhesion of Ph‐positive ALL cell lines through the CD49f‐CD29 heterodimer. Effects of anti‐CD49f (A and B), anti‐CD29 (C and D), and anti‐CD104 (E and F) blocking antibodies or isotype IgG (A, C, andE). Images of three Ph‐positive ALL cell lines attached to laminin‐uncoated (−) or laminin‐coated (+) plates after DAPI staining. (B, D, and F) Comparison of numbers of attached cell nuclei on laminin‐uncoated (−) or laminin‐coated (+) plates. The vertical axis indicates number of attached cell nuclei. The *p*‐value in the paired *t*‐test is indicated at the top when significant.
**Figure S7.** Dephosphorylation of BCR::ABL1 protein in Ph‐positive ALL cell lines treated with imatinib. Four Ph‐positive ALL cell lines were cultured in the presence or absence (control) of 0.5 μM of imatinib for 12 h. Each cell line was also cultured in the presence of 0.01% of DMSO for 12 h as a mock. Immunoblotting was performed with the anti‐ABL (BD Pharmingen #554148, 1:1000 dilution), anti‐Phsoph‐Tyr 204‐cABL (Cell Signaling #C4285, 1:1000 dilution), and anti‐α‐Tubulin (Sigma #T‐5168, 1:1000 dilution) antibodies as previously reported.^28^
**Figure S8.** Cell cycle analyses of Ph‐positive ALL cell lines treated with imatinib. Four Ph‐positive ALL cell lines were cultured in the presence or absence (control) of 0.5 μM of imatinib for 72 h, and, then, cell cycle analyses were performed using the PI staining. Each cell line was also cultured in the presence of 0.01% of DMSO for 72 h as Mock.
**Figure S9.** Downregulation of CD49f expression in Ph‐positive ALL cell lines following imatinib treatment. (A) Flow cytometric analysis of CD49f expression in four Ph‐positive ALL cell lines after 3 and 7 days culture in the presence or absence (Control) of 0.01% DMSO (Mock) or 0.5 μM of imatinib (Imatinib). Red‐filled peaks and blue lines indicate histograms of specific antibody and isotype control, respectively. Representative data in the triplicated analyses are indicated. (B) Downregulation of CD49f expression in four Ph‐positive ALL cell lines after 3 (blue column) and 7 (red column) days incubation with 0.5 μM of imatinib. Vertical axes indicate fold changes in MFI. Median and SE of triplicated analyses are indicated. *p < .05; **p < .01 in the paired *t*‐test.
**Figure S10.** CD29 and CD104 expression in Ph‐positive ALL cell lines treated with imatinib. (A and B) Flow cytometric analysis of CD29 (A) and CD104 (B) expression in four Ph‐positive ALL cell lines after 0 (top panel), 3 (middle panel), and 7 (bottom panel) days of incubation with 0.5 μM of imatinib. Red‐filled peaks and blue lines indicate histograms of specific antibody and isotype control, respectively.
**Figure S11.** Dephosphorylation of BCR::ABL1 protein by the imatinib treatment in Ph‐positive ALL cell lines, but not in their T315I mutated sublines. Two pairs of imatinib‐sensitive parental cell lines (SU‐Ph2 and KOPN55bi) and their T315I‐acquired sublines (SU/SR and KOPN55biR) were cultured in the presence or absence (control) of 0.5 μM of imatinib for 12 h. Each cell line was also cultured in the presence of 0.01% of DMSO for 12 h as Mock. Immunoblotting was performed with the anti‐ABL (BD Pharmingen #554148, 1:1000 dilution), anti‐Phsoph‐Tyr 204‐cABL (Cell Signaling #C4285, 1:1000 dilution), and anti‐α‐Tubulin (Sigma #T‐5168, 1:1000 dilution) antibodies as previously reported.^28^
**Figure S12.** Downregulation of CD49f expression by the imatinib treatment in Ph‐positive ALL cell lines, but not in their T315I mutated sublines. (A) Flow cytometric analysis of CD49f expression in two pairs of imatinib‐sensitive parental cell lines (SU‐Ph2 and KOPN55bi) and their T315I‐acquired sublines (SU/SR and KOPN55biR) after 3 days culture in the presence or absence (Control) of 0.01% DMSO (Mock) or 0.5 μM of imatinib (Imatinib). Red‐filled peaks and blue lines indicate histograms of specific antibody and isotype control, respectively. Representative data in the triplicated analyses are indicated. (B) Downregulation of CD49f expression by the imatinib treatment in Ph‐positive ALL cell lines, but not in their T315I mutated sublines after 3 days incubation with 0.5 μM of (Imatinib). Vertical axes indicate fold changes in MFI. Median and SE of triplicated analyses are indicated. **p < .01 in the paired *t*‐test.
**Figure S13.** CD29 and CD104 expression in Ph‐positive ALL cell lines and their T315I‐acquired sublines treated with imatinib. Flow cytometric analysis of CD29 (A) and CD104 (B) expression in two pairs of imatinib‐sensitive parental cell lines (SU‐Ph2 and KOPN55bi) and their T315I‐acquired sublines (SU/SR and KOPN55biR) after imatinib (0.5 μM for 3 days) treatment. Red‐filled peaks and blue lines indicate histograms of specific antibody and isotype control, respectively.
**Figure S14.** Disruption of laminin‐binding in Ph‐positive ALL cell lines following imatinib treatment. Images of attached cell nuclei of imatinib‐untreated (upper panels) and imatinib‐treated (0.5 μM for 7 days) (lower panels) KOPN55bi (left panels) and KOPN55biR (right panels) cells in the laminin‐coated plates after DAPI staining.

## Data Availability

Data sharing is not applicable to this article as no new data were created or analyzed in this study.
