# Supplementary figures and images for: Involvement of BCR::ABL1 in laminin adhesion of Philadelphia chromosome‐positive acute lymphoblastic leukemia through upregulation of integrin α6
Source: Cancer Rep (Hoboken). 2024 Apr 5;7(4):e2034. doi: 10.1002/cnr2.2034 (PMC10995707; doi:10.1002/cnr2.2034)

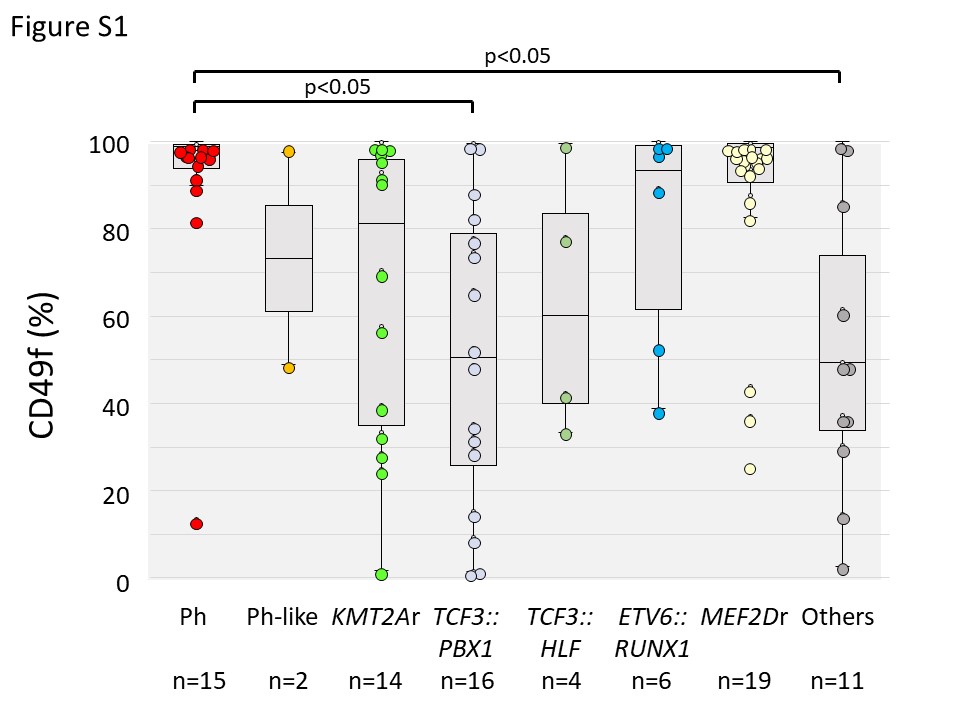

Supplement: Supplementary file 1 — Figure S1. High CD49f expression levels in Ph‐positive ALL. Comparison of cell surface CD49f expression (%; vertical axis) levels in Ph‐positive ALL cell lines with those in each of 6 representative translocations among 87 BCP‐ALL cell lines. The p‐value in the Mann–Whitney analysis is indicated at the top when significant. Figure S2. Correlation between gene and cell surface expression levels of CD49f. Correlation between gene (horizontal axis) and cell surface expression (%; vertical axis) levels of CD49f in 27 BCP‐ALL cell lines, including 6 Ph‐positive ALL cell lines (red circles). The correlation coefficient is indicated at the top. Figure S3. CD29 and CD104 gene expression levels in Ph‐positive ALL samples. (A and B) Association of representative chromosomal abnormalities with CD29 gene expression (vertical axis) levels in childhood BCP‐ALL samples of the NOPHO (A) and St. Jude (B) cohorts. (C and D) Association of representative chromosomal abnormalities with CD104 gene expression (vertical axis) levels in childhood BCP‐ALL samples of the NOPHO (C) and St. Jude (D) cohorts. Figure S4. Laminin adhesion of Ph‐positive ALL cell lines. Images of three Ph‐positive ALL cell lines (KOPN30bi, KOPN55bi, KOPN66bi) attached to laminin‐uncoated (−) (upper panel) or laminin‐coated (+) (lower panel) plates after DAPI staining. Figure S5. Experimental workflow of blocking assay of laminin adhesion by specific antibodies. Figure S6. Laminin‐adhesion of Ph‐positive ALL cell lines through the CD49f‐CD29 heterodimer. Effects of anti‐CD49f (A and B), anti‐CD29 (C and D), and anti‐CD104 (E and F) blocking antibodies or isotype IgG (A, C, andE). Images of three Ph‐positive ALL cell lines attached to laminin‐uncoated (−) or laminin‐coated (+) plates after DAPI staining. (B, D, and F) Comparison of numbers of attached cell nuclei on laminin‐uncoated (−) or laminin‐coated (+) plates. The vertical axis indicates number of attached cell nuclei. The p‐value in the paired t‐test is indic [file CNR2-7-e2034-s001.zip › Figure S1.jpg]

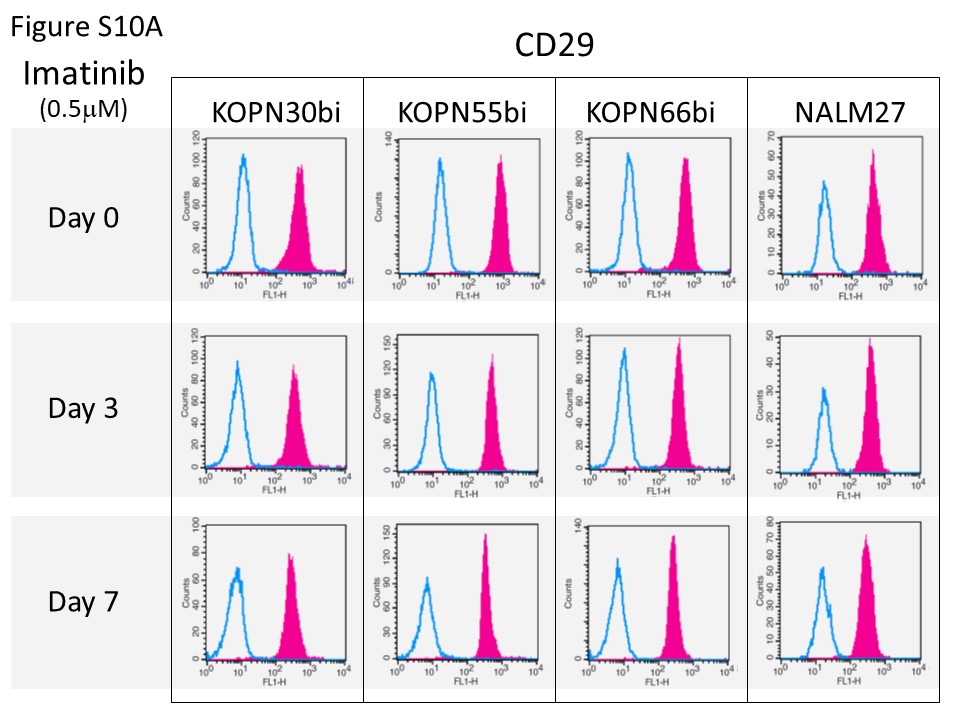

Supplement: Supplementary file 1 — Figure S1. High CD49f expression levels in Ph‐positive ALL. Comparison of cell surface CD49f expression (%; vertical axis) levels in Ph‐positive ALL cell lines with those in each of 6 representative translocations among 87 BCP‐ALL cell lines. The p‐value in the Mann–Whitney analysis is indicated at the top when significant. Figure S2. Correlation between gene and cell surface expression levels of CD49f. Correlation between gene (horizontal axis) and cell surface expression (%; vertical axis) levels of CD49f in 27 BCP‐ALL cell lines, including 6 Ph‐positive ALL cell lines (red circles). The correlation coefficient is indicated at the top. Figure S3. CD29 and CD104 gene expression levels in Ph‐positive ALL samples. (A and B) Association of representative chromosomal abnormalities with CD29 gene expression (vertical axis) levels in childhood BCP‐ALL samples of the NOPHO (A) and St. Jude (B) cohorts. (C and D) Association of representative chromosomal abnormalities with CD104 gene expression (vertical axis) levels in childhood BCP‐ALL samples of the NOPHO (C) and St. Jude (D) cohorts. Figure S4. Laminin adhesion of Ph‐positive ALL cell lines. Images of three Ph‐positive ALL cell lines (KOPN30bi, KOPN55bi, KOPN66bi) attached to laminin‐uncoated (−) (upper panel) or laminin‐coated (+) (lower panel) plates after DAPI staining. Figure S5. Experimental workflow of blocking assay of laminin adhesion by specific antibodies. Figure S6. Laminin‐adhesion of Ph‐positive ALL cell lines through the CD49f‐CD29 heterodimer. Effects of anti‐CD49f (A and B), anti‐CD29 (C and D), and anti‐CD104 (E and F) blocking antibodies or isotype IgG (A, C, andE). Images of three Ph‐positive ALL cell lines attached to laminin‐uncoated (−) or laminin‐coated (+) plates after DAPI staining. (B, D, and F) Comparison of numbers of attached cell nuclei on laminin‐uncoated (−) or laminin‐coated (+) plates. The vertical axis indicates number of attached cell nuclei. The p‐value in the paired t‐test is indic [file CNR2-7-e2034-s001.zip › Figure S10A.jpg]

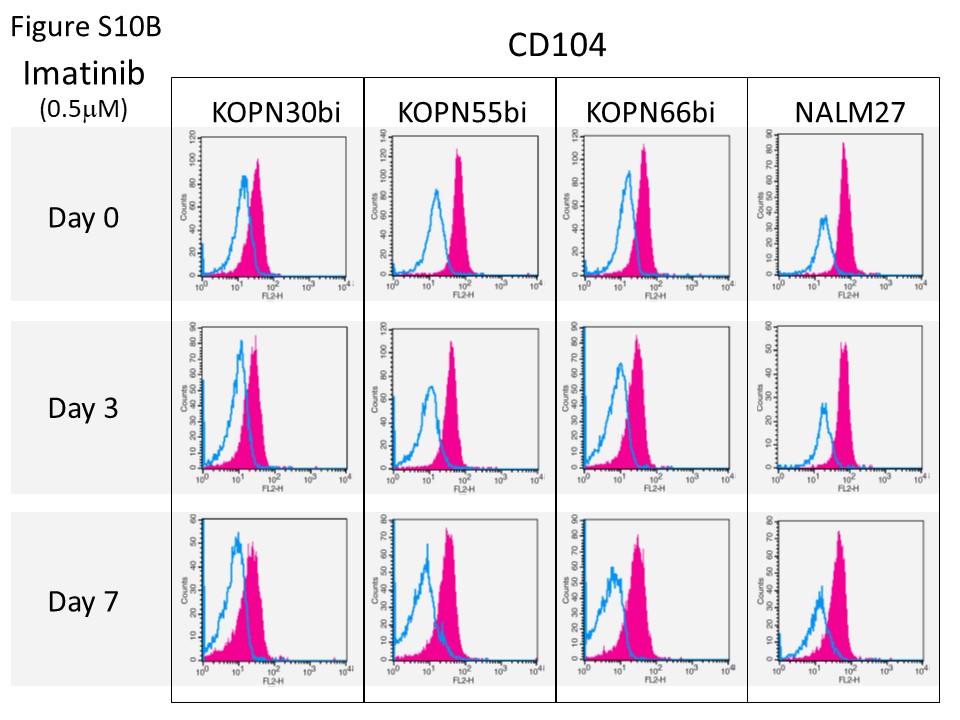

Supplement: Supplementary file 1 — Figure S1. High CD49f expression levels in Ph‐positive ALL. Comparison of cell surface CD49f expression (%; vertical axis) levels in Ph‐positive ALL cell lines with those in each of 6 representative translocations among 87 BCP‐ALL cell lines. The p‐value in the Mann–Whitney analysis is indicated at the top when significant. Figure S2. Correlation between gene and cell surface expression levels of CD49f. Correlation between gene (horizontal axis) and cell surface expression (%; vertical axis) levels of CD49f in 27 BCP‐ALL cell lines, including 6 Ph‐positive ALL cell lines (red circles). The correlation coefficient is indicated at the top. Figure S3. CD29 and CD104 gene expression levels in Ph‐positive ALL samples. (A and B) Association of representative chromosomal abnormalities with CD29 gene expression (vertical axis) levels in childhood BCP‐ALL samples of the NOPHO (A) and St. Jude (B) cohorts. (C and D) Association of representative chromosomal abnormalities with CD104 gene expression (vertical axis) levels in childhood BCP‐ALL samples of the NOPHO (C) and St. Jude (D) cohorts. Figure S4. Laminin adhesion of Ph‐positive ALL cell lines. Images of three Ph‐positive ALL cell lines (KOPN30bi, KOPN55bi, KOPN66bi) attached to laminin‐uncoated (−) (upper panel) or laminin‐coated (+) (lower panel) plates after DAPI staining. Figure S5. Experimental workflow of blocking assay of laminin adhesion by specific antibodies. Figure S6. Laminin‐adhesion of Ph‐positive ALL cell lines through the CD49f‐CD29 heterodimer. Effects of anti‐CD49f (A and B), anti‐CD29 (C and D), and anti‐CD104 (E and F) blocking antibodies or isotype IgG (A, C, andE). Images of three Ph‐positive ALL cell lines attached to laminin‐uncoated (−) or laminin‐coated (+) plates after DAPI staining. (B, D, and F) Comparison of numbers of attached cell nuclei on laminin‐uncoated (−) or laminin‐coated (+) plates. The vertical axis indicates number of attached cell nuclei. The p‐value in the paired t‐test is indic [file CNR2-7-e2034-s001.zip › Figure S10B.jpg]

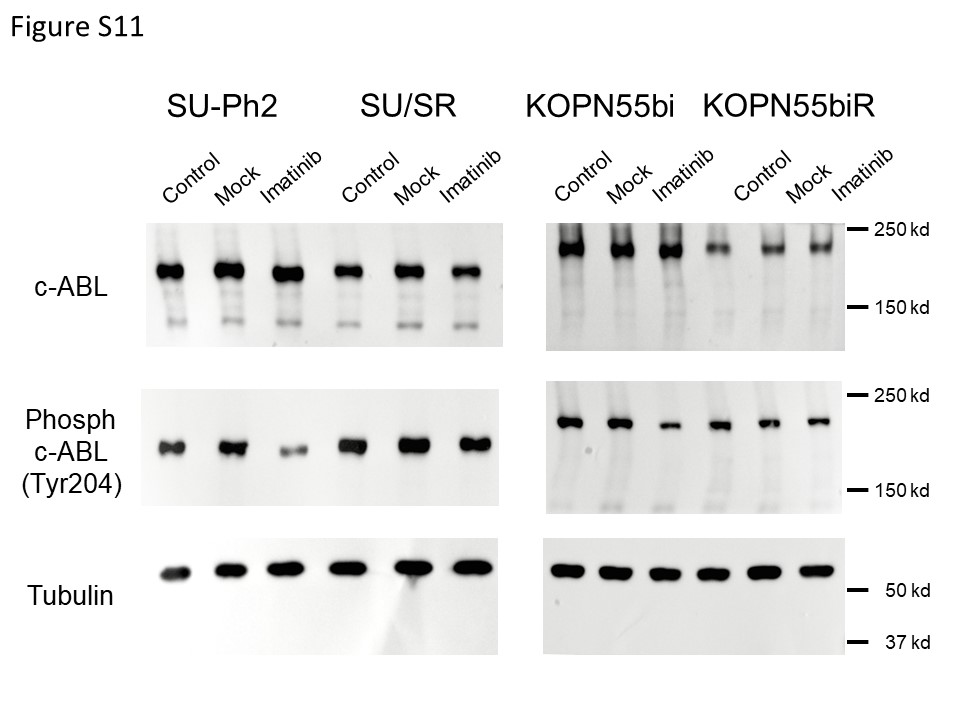

Supplement: Supplementary file 1 — Figure S1. High CD49f expression levels in Ph‐positive ALL. Comparison of cell surface CD49f expression (%; vertical axis) levels in Ph‐positive ALL cell lines with those in each of 6 representative translocations among 87 BCP‐ALL cell lines. The p‐value in the Mann–Whitney analysis is indicated at the top when significant. Figure S2. Correlation between gene and cell surface expression levels of CD49f. Correlation between gene (horizontal axis) and cell surface expression (%; vertical axis) levels of CD49f in 27 BCP‐ALL cell lines, including 6 Ph‐positive ALL cell lines (red circles). The correlation coefficient is indicated at the top. Figure S3. CD29 and CD104 gene expression levels in Ph‐positive ALL samples. (A and B) Association of representative chromosomal abnormalities with CD29 gene expression (vertical axis) levels in childhood BCP‐ALL samples of the NOPHO (A) and St. Jude (B) cohorts. (C and D) Association of representative chromosomal abnormalities with CD104 gene expression (vertical axis) levels in childhood BCP‐ALL samples of the NOPHO (C) and St. Jude (D) cohorts. Figure S4. Laminin adhesion of Ph‐positive ALL cell lines. Images of three Ph‐positive ALL cell lines (KOPN30bi, KOPN55bi, KOPN66bi) attached to laminin‐uncoated (−) (upper panel) or laminin‐coated (+) (lower panel) plates after DAPI staining. Figure S5. Experimental workflow of blocking assay of laminin adhesion by specific antibodies. Figure S6. Laminin‐adhesion of Ph‐positive ALL cell lines through the CD49f‐CD29 heterodimer. Effects of anti‐CD49f (A and B), anti‐CD29 (C and D), and anti‐CD104 (E and F) blocking antibodies or isotype IgG (A, C, andE). Images of three Ph‐positive ALL cell lines attached to laminin‐uncoated (−) or laminin‐coated (+) plates after DAPI staining. (B, D, and F) Comparison of numbers of attached cell nuclei on laminin‐uncoated (−) or laminin‐coated (+) plates. The vertical axis indicates number of attached cell nuclei. The p‐value in the paired t‐test is indic [file CNR2-7-e2034-s001.zip › Figure S11.jpg]

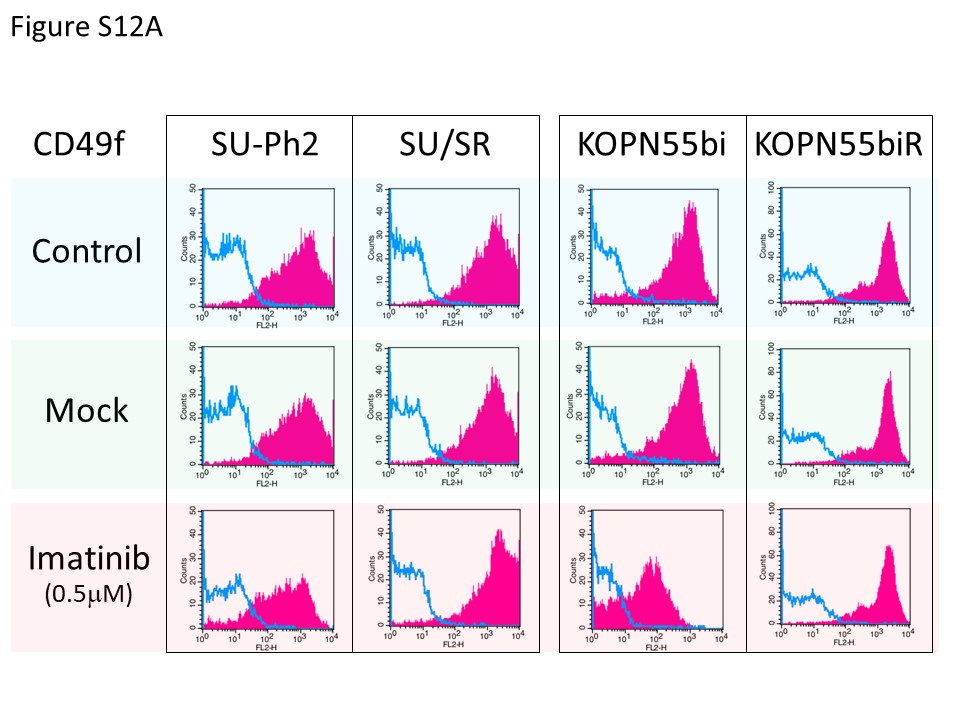

Supplement: Supplementary file 1 — Figure S1. High CD49f expression levels in Ph‐positive ALL. Comparison of cell surface CD49f expression (%; vertical axis) levels in Ph‐positive ALL cell lines with those in each of 6 representative translocations among 87 BCP‐ALL cell lines. The p‐value in the Mann–Whitney analysis is indicated at the top when significant. Figure S2. Correlation between gene and cell surface expression levels of CD49f. Correlation between gene (horizontal axis) and cell surface expression (%; vertical axis) levels of CD49f in 27 BCP‐ALL cell lines, including 6 Ph‐positive ALL cell lines (red circles). The correlation coefficient is indicated at the top. Figure S3. CD29 and CD104 gene expression levels in Ph‐positive ALL samples. (A and B) Association of representative chromosomal abnormalities with CD29 gene expression (vertical axis) levels in childhood BCP‐ALL samples of the NOPHO (A) and St. Jude (B) cohorts. (C and D) Association of representative chromosomal abnormalities with CD104 gene expression (vertical axis) levels in childhood BCP‐ALL samples of the NOPHO (C) and St. Jude (D) cohorts. Figure S4. Laminin adhesion of Ph‐positive ALL cell lines. Images of three Ph‐positive ALL cell lines (KOPN30bi, KOPN55bi, KOPN66bi) attached to laminin‐uncoated (−) (upper panel) or laminin‐coated (+) (lower panel) plates after DAPI staining. Figure S5. Experimental workflow of blocking assay of laminin adhesion by specific antibodies. Figure S6. Laminin‐adhesion of Ph‐positive ALL cell lines through the CD49f‐CD29 heterodimer. Effects of anti‐CD49f (A and B), anti‐CD29 (C and D), and anti‐CD104 (E and F) blocking antibodies or isotype IgG (A, C, andE). Images of three Ph‐positive ALL cell lines attached to laminin‐uncoated (−) or laminin‐coated (+) plates after DAPI staining. (B, D, and F) Comparison of numbers of attached cell nuclei on laminin‐uncoated (−) or laminin‐coated (+) plates. The vertical axis indicates number of attached cell nuclei. The p‐value in the paired t‐test is indic [file CNR2-7-e2034-s001.zip › Figure S12A.jpg]

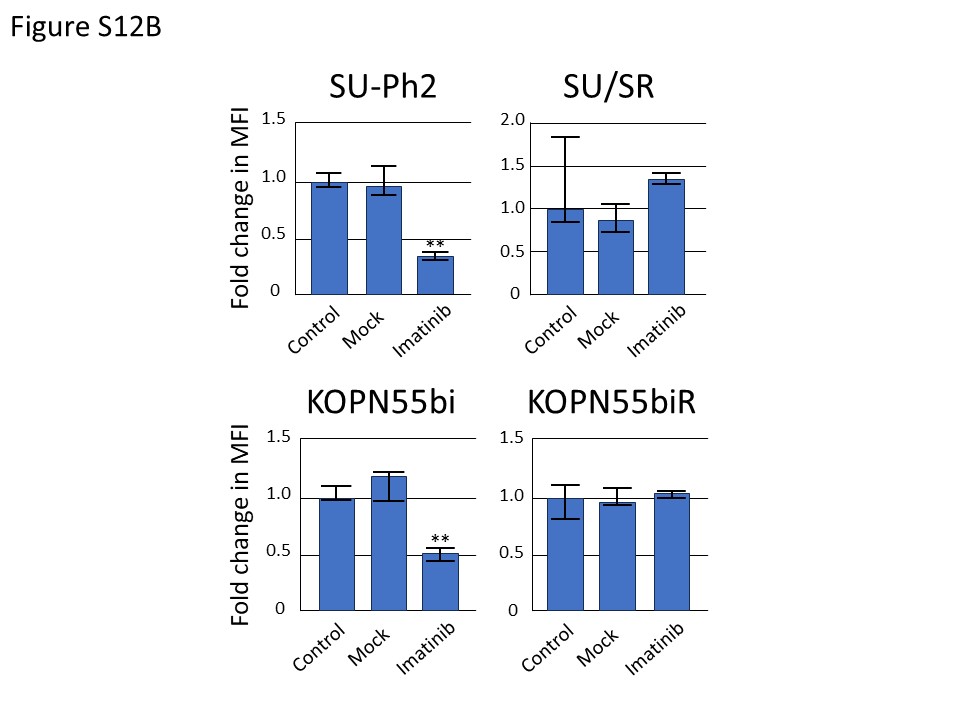

Supplement: Supplementary file 1 — Figure S1. High CD49f expression levels in Ph‐positive ALL. Comparison of cell surface CD49f expression (%; vertical axis) levels in Ph‐positive ALL cell lines with those in each of 6 representative translocations among 87 BCP‐ALL cell lines. The p‐value in the Mann–Whitney analysis is indicated at the top when significant. Figure S2. Correlation between gene and cell surface expression levels of CD49f. Correlation between gene (horizontal axis) and cell surface expression (%; vertical axis) levels of CD49f in 27 BCP‐ALL cell lines, including 6 Ph‐positive ALL cell lines (red circles). The correlation coefficient is indicated at the top. Figure S3. CD29 and CD104 gene expression levels in Ph‐positive ALL samples. (A and B) Association of representative chromosomal abnormalities with CD29 gene expression (vertical axis) levels in childhood BCP‐ALL samples of the NOPHO (A) and St. Jude (B) cohorts. (C and D) Association of representative chromosomal abnormalities with CD104 gene expression (vertical axis) levels in childhood BCP‐ALL samples of the NOPHO (C) and St. Jude (D) cohorts. Figure S4. Laminin adhesion of Ph‐positive ALL cell lines. Images of three Ph‐positive ALL cell lines (KOPN30bi, KOPN55bi, KOPN66bi) attached to laminin‐uncoated (−) (upper panel) or laminin‐coated (+) (lower panel) plates after DAPI staining. Figure S5. Experimental workflow of blocking assay of laminin adhesion by specific antibodies. Figure S6. Laminin‐adhesion of Ph‐positive ALL cell lines through the CD49f‐CD29 heterodimer. Effects of anti‐CD49f (A and B), anti‐CD29 (C and D), and anti‐CD104 (E and F) blocking antibodies or isotype IgG (A, C, andE). Images of three Ph‐positive ALL cell lines attached to laminin‐uncoated (−) or laminin‐coated (+) plates after DAPI staining. (B, D, and F) Comparison of numbers of attached cell nuclei on laminin‐uncoated (−) or laminin‐coated (+) plates. The vertical axis indicates number of attached cell nuclei. The p‐value in the paired t‐test is indic [file CNR2-7-e2034-s001.zip › Figure S12B.jpg]

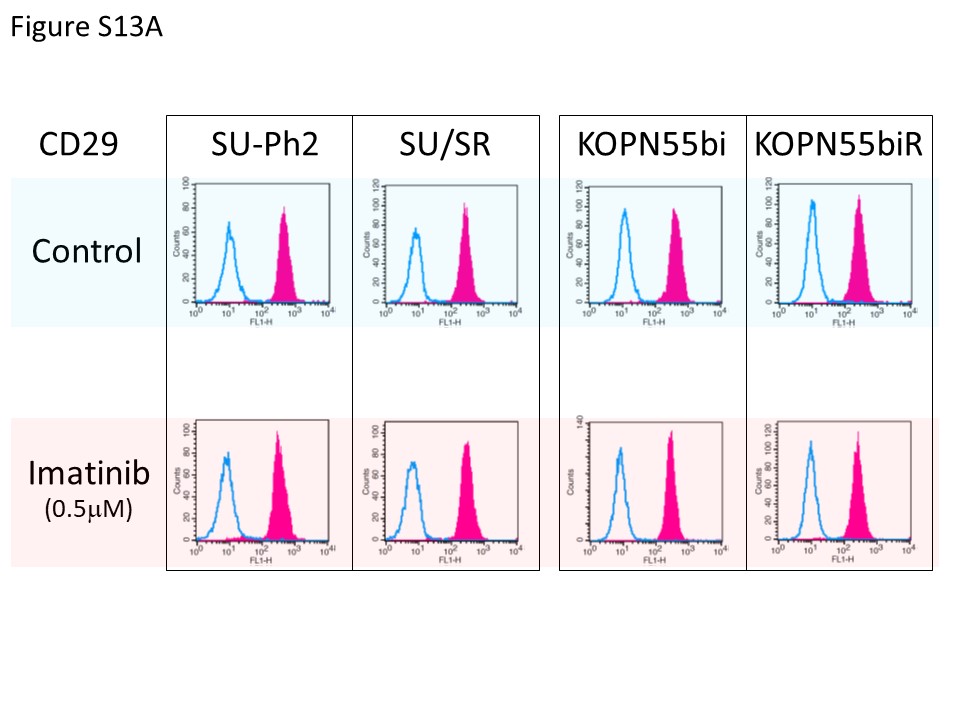

Supplement: Supplementary file 1 — Figure S1. High CD49f expression levels in Ph‐positive ALL. Comparison of cell surface CD49f expression (%; vertical axis) levels in Ph‐positive ALL cell lines with those in each of 6 representative translocations among 87 BCP‐ALL cell lines. The p‐value in the Mann–Whitney analysis is indicated at the top when significant. Figure S2. Correlation between gene and cell surface expression levels of CD49f. Correlation between gene (horizontal axis) and cell surface expression (%; vertical axis) levels of CD49f in 27 BCP‐ALL cell lines, including 6 Ph‐positive ALL cell lines (red circles). The correlation coefficient is indicated at the top. Figure S3. CD29 and CD104 gene expression levels in Ph‐positive ALL samples. (A and B) Association of representative chromosomal abnormalities with CD29 gene expression (vertical axis) levels in childhood BCP‐ALL samples of the NOPHO (A) and St. Jude (B) cohorts. (C and D) Association of representative chromosomal abnormalities with CD104 gene expression (vertical axis) levels in childhood BCP‐ALL samples of the NOPHO (C) and St. Jude (D) cohorts. Figure S4. Laminin adhesion of Ph‐positive ALL cell lines. Images of three Ph‐positive ALL cell lines (KOPN30bi, KOPN55bi, KOPN66bi) attached to laminin‐uncoated (−) (upper panel) or laminin‐coated (+) (lower panel) plates after DAPI staining. Figure S5. Experimental workflow of blocking assay of laminin adhesion by specific antibodies. Figure S6. Laminin‐adhesion of Ph‐positive ALL cell lines through the CD49f‐CD29 heterodimer. Effects of anti‐CD49f (A and B), anti‐CD29 (C and D), and anti‐CD104 (E and F) blocking antibodies or isotype IgG (A, C, andE). Images of three Ph‐positive ALL cell lines attached to laminin‐uncoated (−) or laminin‐coated (+) plates after DAPI staining. (B, D, and F) Comparison of numbers of attached cell nuclei on laminin‐uncoated (−) or laminin‐coated (+) plates. The vertical axis indicates number of attached cell nuclei. The p‐value in the paired t‐test is indic [file CNR2-7-e2034-s001.zip › Figure S13A.jpg]

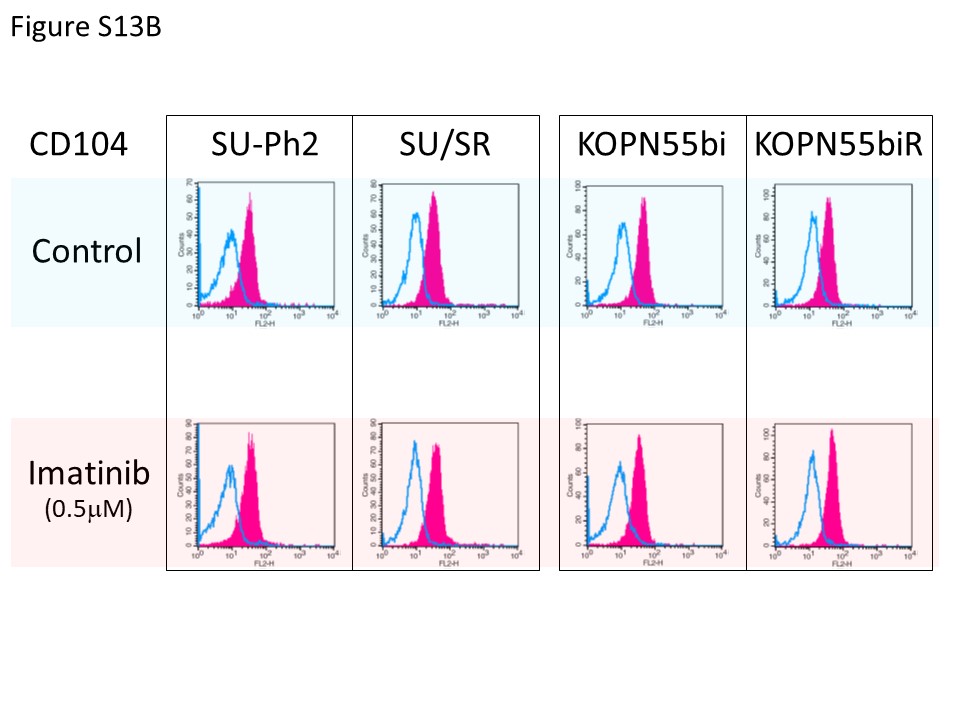

Supplement: Supplementary file 1 — Figure S1. High CD49f expression levels in Ph‐positive ALL. Comparison of cell surface CD49f expression (%; vertical axis) levels in Ph‐positive ALL cell lines with those in each of 6 representative translocations among 87 BCP‐ALL cell lines. The p‐value in the Mann–Whitney analysis is indicated at the top when significant. Figure S2. Correlation between gene and cell surface expression levels of CD49f. Correlation between gene (horizontal axis) and cell surface expression (%; vertical axis) levels of CD49f in 27 BCP‐ALL cell lines, including 6 Ph‐positive ALL cell lines (red circles). The correlation coefficient is indicated at the top. Figure S3. CD29 and CD104 gene expression levels in Ph‐positive ALL samples. (A and B) Association of representative chromosomal abnormalities with CD29 gene expression (vertical axis) levels in childhood BCP‐ALL samples of the NOPHO (A) and St. Jude (B) cohorts. (C and D) Association of representative chromosomal abnormalities with CD104 gene expression (vertical axis) levels in childhood BCP‐ALL samples of the NOPHO (C) and St. Jude (D) cohorts. Figure S4. Laminin adhesion of Ph‐positive ALL cell lines. Images of three Ph‐positive ALL cell lines (KOPN30bi, KOPN55bi, KOPN66bi) attached to laminin‐uncoated (−) (upper panel) or laminin‐coated (+) (lower panel) plates after DAPI staining. Figure S5. Experimental workflow of blocking assay of laminin adhesion by specific antibodies. Figure S6. Laminin‐adhesion of Ph‐positive ALL cell lines through the CD49f‐CD29 heterodimer. Effects of anti‐CD49f (A and B), anti‐CD29 (C and D), and anti‐CD104 (E and F) blocking antibodies or isotype IgG (A, C, andE). Images of three Ph‐positive ALL cell lines attached to laminin‐uncoated (−) or laminin‐coated (+) plates after DAPI staining. (B, D, and F) Comparison of numbers of attached cell nuclei on laminin‐uncoated (−) or laminin‐coated (+) plates. The vertical axis indicates number of attached cell nuclei. The p‐value in the paired t‐test is indic [file CNR2-7-e2034-s001.zip › Figure S13B.jpg]

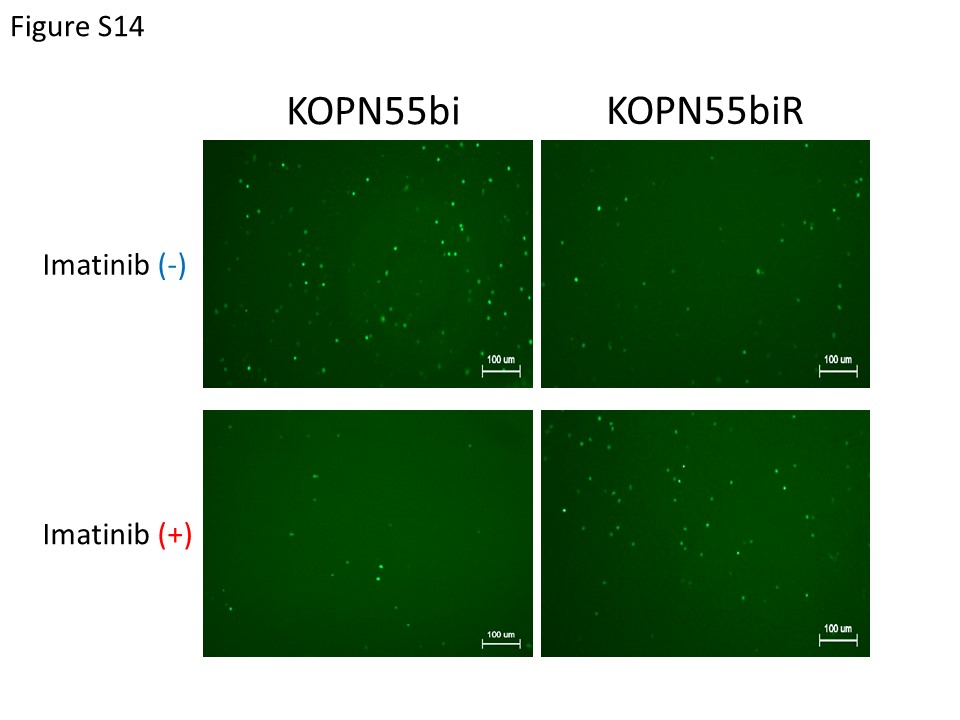

Supplement: Supplementary file 1 — Figure S1. High CD49f expression levels in Ph‐positive ALL. Comparison of cell surface CD49f expression (%; vertical axis) levels in Ph‐positive ALL cell lines with those in each of 6 representative translocations among 87 BCP‐ALL cell lines. The p‐value in the Mann–Whitney analysis is indicated at the top when significant. Figure S2. Correlation between gene and cell surface expression levels of CD49f. Correlation between gene (horizontal axis) and cell surface expression (%; vertical axis) levels of CD49f in 27 BCP‐ALL cell lines, including 6 Ph‐positive ALL cell lines (red circles). The correlation coefficient is indicated at the top. Figure S3. CD29 and CD104 gene expression levels in Ph‐positive ALL samples. (A and B) Association of representative chromosomal abnormalities with CD29 gene expression (vertical axis) levels in childhood BCP‐ALL samples of the NOPHO (A) and St. Jude (B) cohorts. (C and D) Association of representative chromosomal abnormalities with CD104 gene expression (vertical axis) levels in childhood BCP‐ALL samples of the NOPHO (C) and St. Jude (D) cohorts. Figure S4. Laminin adhesion of Ph‐positive ALL cell lines. Images of three Ph‐positive ALL cell lines (KOPN30bi, KOPN55bi, KOPN66bi) attached to laminin‐uncoated (−) (upper panel) or laminin‐coated (+) (lower panel) plates after DAPI staining. Figure S5. Experimental workflow of blocking assay of laminin adhesion by specific antibodies. Figure S6. Laminin‐adhesion of Ph‐positive ALL cell lines through the CD49f‐CD29 heterodimer. Effects of anti‐CD49f (A and B), anti‐CD29 (C and D), and anti‐CD104 (E and F) blocking antibodies or isotype IgG (A, C, andE). Images of three Ph‐positive ALL cell lines attached to laminin‐uncoated (−) or laminin‐coated (+) plates after DAPI staining. (B, D, and F) Comparison of numbers of attached cell nuclei on laminin‐uncoated (−) or laminin‐coated (+) plates. The vertical axis indicates number of attached cell nuclei. The p‐value in the paired t‐test is indic [file CNR2-7-e2034-s001.zip › Figure S14.jpg]

Figure S2

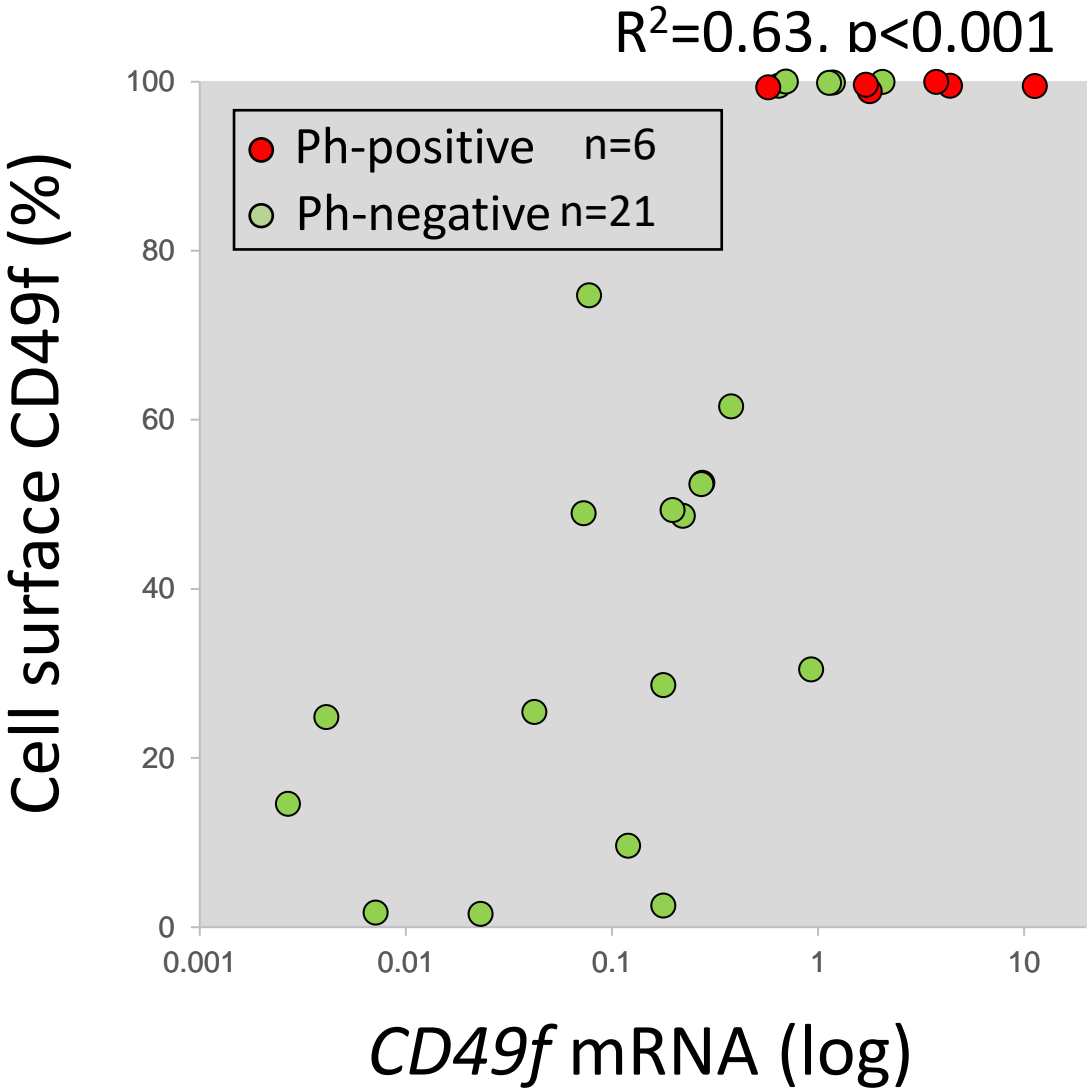

Supplement: Supplementary file 1 — Figure S1. High CD49f expression levels in Ph‐positive ALL. Comparison of cell surface CD49f expression (%; vertical axis) levels in Ph‐positive ALL cell lines with those in each of 6 representative translocations among 87 BCP‐ALL cell lines. The p‐value in the Mann–Whitney analysis is indicated at the top when significant. Figure S2. Correlation between gene and cell surface expression levels of CD49f. Correlation between gene (horizontal axis) and cell surface expression (%; vertical axis) levels of CD49f in 27 BCP‐ALL cell lines, including 6 Ph‐positive ALL cell lines (red circles). The correlation coefficient is indicated at the top. Figure S3. CD29 and CD104 gene expression levels in Ph‐positive ALL samples. (A and B) Association of representative chromosomal abnormalities with CD29 gene expression (vertical axis) levels in childhood BCP‐ALL samples of the NOPHO (A) and St. Jude (B) cohorts. (C and D) Association of representative chromosomal abnormalities with CD104 gene expression (vertical axis) levels in childhood BCP‐ALL samples of the NOPHO (C) and St. Jude (D) cohorts. Figure S4. Laminin adhesion of Ph‐positive ALL cell lines. Images of three Ph‐positive ALL cell lines (KOPN30bi, KOPN55bi, KOPN66bi) attached to laminin‐uncoated (−) (upper panel) or laminin‐coated (+) (lower panel) plates after DAPI staining. Figure S5. Experimental workflow of blocking assay of laminin adhesion by specific antibodies. Figure S6. Laminin‐adhesion of Ph‐positive ALL cell lines through the CD49f‐CD29 heterodimer. Effects of anti‐CD49f (A and B), anti‐CD29 (C and D), and anti‐CD104 (E and F) blocking antibodies or isotype IgG (A, C, andE). Images of three Ph‐positive ALL cell lines attached to laminin‐uncoated (−) or laminin‐coated (+) plates after DAPI staining. (B, D, and F) Comparison of numbers of attached cell nuclei on laminin‐uncoated (−) or laminin‐coated (+) plates. The vertical axis indicates number of attached cell nuclei. The p‐value in the paired t‐test is indic [file CNR2-7-e2034-s001.zip › Figure S2.pdf]

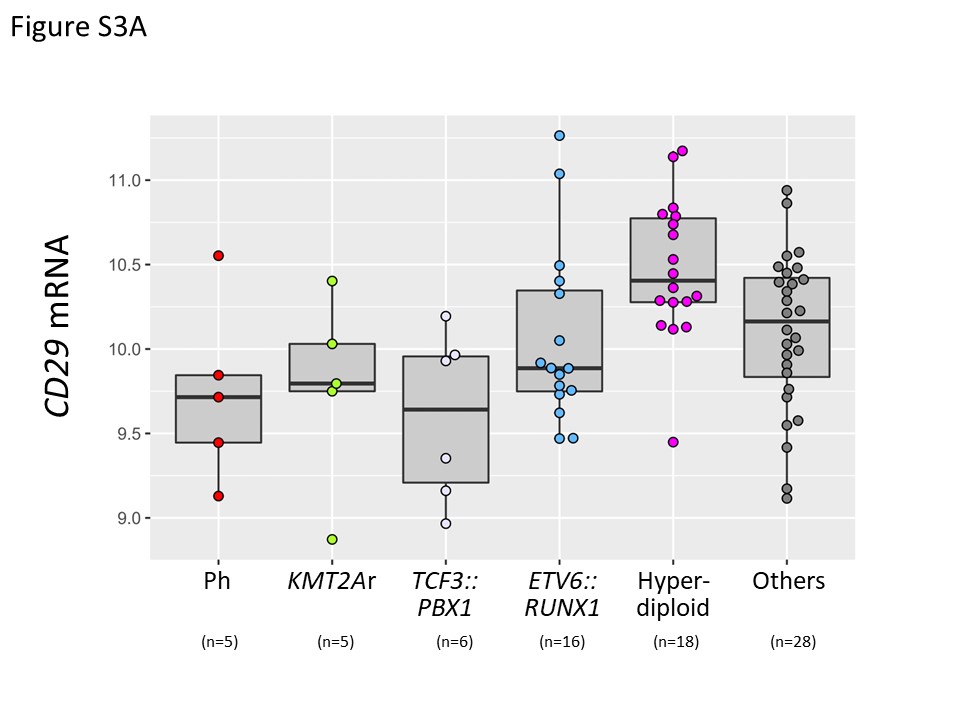

Supplement: Supplementary file 1 — Figure S1. High CD49f expression levels in Ph‐positive ALL. Comparison of cell surface CD49f expression (%; vertical axis) levels in Ph‐positive ALL cell lines with those in each of 6 representative translocations among 87 BCP‐ALL cell lines. The p‐value in the Mann–Whitney analysis is indicated at the top when significant. Figure S2. Correlation between gene and cell surface expression levels of CD49f. Correlation between gene (horizontal axis) and cell surface expression (%; vertical axis) levels of CD49f in 27 BCP‐ALL cell lines, including 6 Ph‐positive ALL cell lines (red circles). The correlation coefficient is indicated at the top. Figure S3. CD29 and CD104 gene expression levels in Ph‐positive ALL samples. (A and B) Association of representative chromosomal abnormalities with CD29 gene expression (vertical axis) levels in childhood BCP‐ALL samples of the NOPHO (A) and St. Jude (B) cohorts. (C and D) Association of representative chromosomal abnormalities with CD104 gene expression (vertical axis) levels in childhood BCP‐ALL samples of the NOPHO (C) and St. Jude (D) cohorts. Figure S4. Laminin adhesion of Ph‐positive ALL cell lines. Images of three Ph‐positive ALL cell lines (KOPN30bi, KOPN55bi, KOPN66bi) attached to laminin‐uncoated (−) (upper panel) or laminin‐coated (+) (lower panel) plates after DAPI staining. Figure S5. Experimental workflow of blocking assay of laminin adhesion by specific antibodies. Figure S6. Laminin‐adhesion of Ph‐positive ALL cell lines through the CD49f‐CD29 heterodimer. Effects of anti‐CD49f (A and B), anti‐CD29 (C and D), and anti‐CD104 (E and F) blocking antibodies or isotype IgG (A, C, andE). Images of three Ph‐positive ALL cell lines attached to laminin‐uncoated (−) or laminin‐coated (+) plates after DAPI staining. (B, D, and F) Comparison of numbers of attached cell nuclei on laminin‐uncoated (−) or laminin‐coated (+) plates. The vertical axis indicates number of attached cell nuclei. The p‐value in the paired t‐test is indic [file CNR2-7-e2034-s001.zip › Figure S3A.jpg]

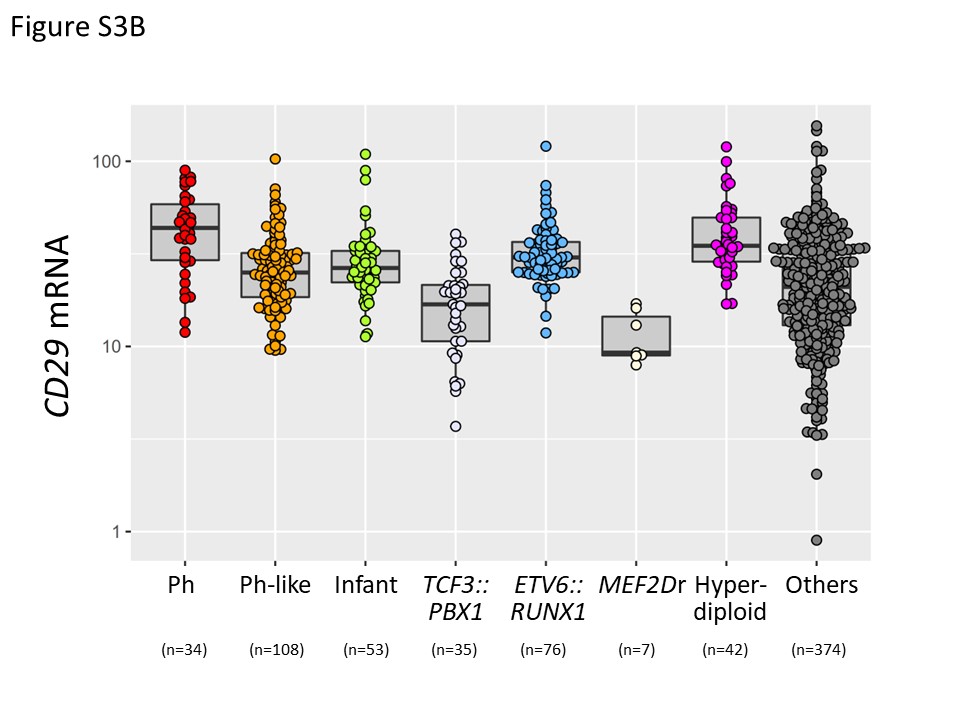

Supplement: Supplementary file 1 — Figure S1. High CD49f expression levels in Ph‐positive ALL. Comparison of cell surface CD49f expression (%; vertical axis) levels in Ph‐positive ALL cell lines with those in each of 6 representative translocations among 87 BCP‐ALL cell lines. The p‐value in the Mann–Whitney analysis is indicated at the top when significant. Figure S2. Correlation between gene and cell surface expression levels of CD49f. Correlation between gene (horizontal axis) and cell surface expression (%; vertical axis) levels of CD49f in 27 BCP‐ALL cell lines, including 6 Ph‐positive ALL cell lines (red circles). The correlation coefficient is indicated at the top. Figure S3. CD29 and CD104 gene expression levels in Ph‐positive ALL samples. (A and B) Association of representative chromosomal abnormalities with CD29 gene expression (vertical axis) levels in childhood BCP‐ALL samples of the NOPHO (A) and St. Jude (B) cohorts. (C and D) Association of representative chromosomal abnormalities with CD104 gene expression (vertical axis) levels in childhood BCP‐ALL samples of the NOPHO (C) and St. Jude (D) cohorts. Figure S4. Laminin adhesion of Ph‐positive ALL cell lines. Images of three Ph‐positive ALL cell lines (KOPN30bi, KOPN55bi, KOPN66bi) attached to laminin‐uncoated (−) (upper panel) or laminin‐coated (+) (lower panel) plates after DAPI staining. Figure S5. Experimental workflow of blocking assay of laminin adhesion by specific antibodies. Figure S6. Laminin‐adhesion of Ph‐positive ALL cell lines through the CD49f‐CD29 heterodimer. Effects of anti‐CD49f (A and B), anti‐CD29 (C and D), and anti‐CD104 (E and F) blocking antibodies or isotype IgG (A, C, andE). Images of three Ph‐positive ALL cell lines attached to laminin‐uncoated (−) or laminin‐coated (+) plates after DAPI staining. (B, D, and F) Comparison of numbers of attached cell nuclei on laminin‐uncoated (−) or laminin‐coated (+) plates. The vertical axis indicates number of attached cell nuclei. The p‐value in the paired t‐test is indic [file CNR2-7-e2034-s001.zip › Figure S3B.jpg]

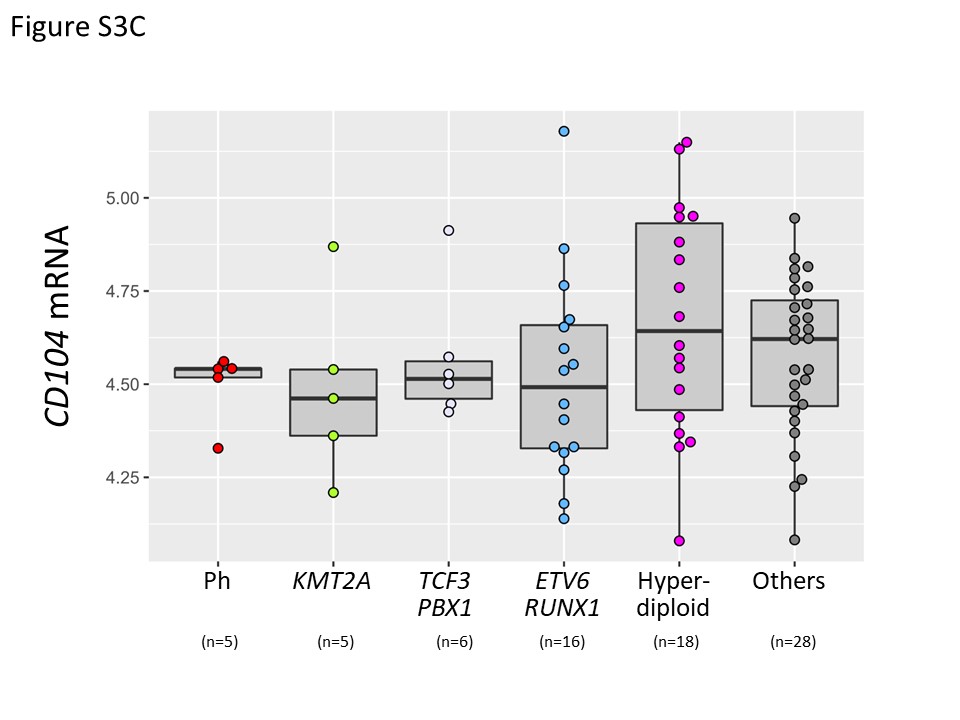

Supplement: Supplementary file 1 — Figure S1. High CD49f expression levels in Ph‐positive ALL. Comparison of cell surface CD49f expression (%; vertical axis) levels in Ph‐positive ALL cell lines with those in each of 6 representative translocations among 87 BCP‐ALL cell lines. The p‐value in the Mann–Whitney analysis is indicated at the top when significant. Figure S2. Correlation between gene and cell surface expression levels of CD49f. Correlation between gene (horizontal axis) and cell surface expression (%; vertical axis) levels of CD49f in 27 BCP‐ALL cell lines, including 6 Ph‐positive ALL cell lines (red circles). The correlation coefficient is indicated at the top. Figure S3. CD29 and CD104 gene expression levels in Ph‐positive ALL samples. (A and B) Association of representative chromosomal abnormalities with CD29 gene expression (vertical axis) levels in childhood BCP‐ALL samples of the NOPHO (A) and St. Jude (B) cohorts. (C and D) Association of representative chromosomal abnormalities with CD104 gene expression (vertical axis) levels in childhood BCP‐ALL samples of the NOPHO (C) and St. Jude (D) cohorts. Figure S4. Laminin adhesion of Ph‐positive ALL cell lines. Images of three Ph‐positive ALL cell lines (KOPN30bi, KOPN55bi, KOPN66bi) attached to laminin‐uncoated (−) (upper panel) or laminin‐coated (+) (lower panel) plates after DAPI staining. Figure S5. Experimental workflow of blocking assay of laminin adhesion by specific antibodies. Figure S6. Laminin‐adhesion of Ph‐positive ALL cell lines through the CD49f‐CD29 heterodimer. Effects of anti‐CD49f (A and B), anti‐CD29 (C and D), and anti‐CD104 (E and F) blocking antibodies or isotype IgG (A, C, andE). Images of three Ph‐positive ALL cell lines attached to laminin‐uncoated (−) or laminin‐coated (+) plates after DAPI staining. (B, D, and F) Comparison of numbers of attached cell nuclei on laminin‐uncoated (−) or laminin‐coated (+) plates. The vertical axis indicates number of attached cell nuclei. The p‐value in the paired t‐test is indic [file CNR2-7-e2034-s001.zip › Figure S3C.jpg]

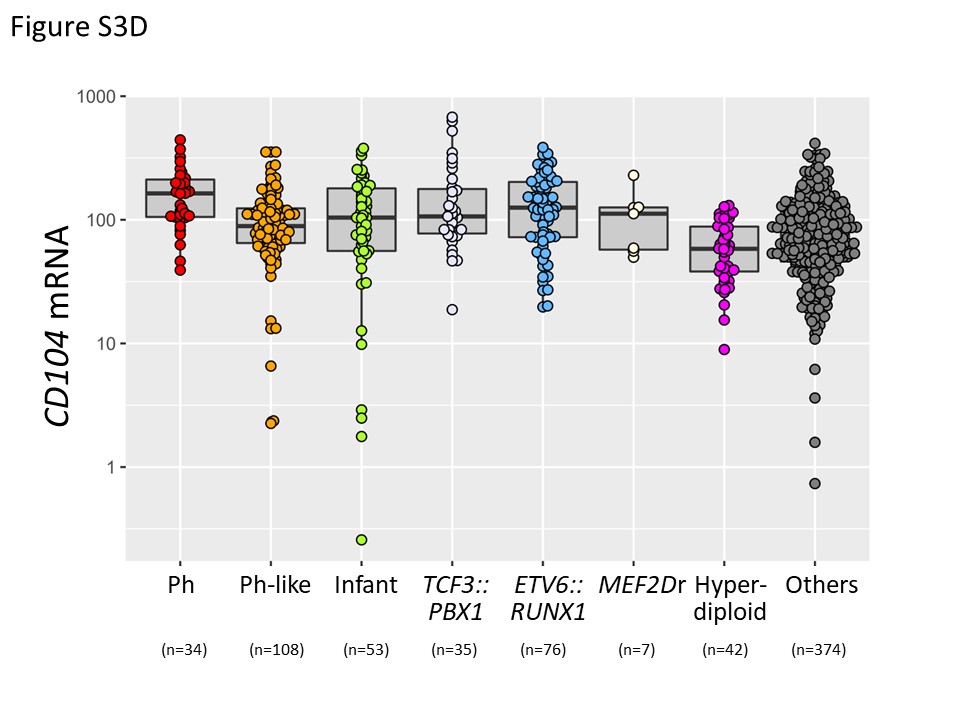

Supplement: Supplementary file 1 — Figure S1. High CD49f expression levels in Ph‐positive ALL. Comparison of cell surface CD49f expression (%; vertical axis) levels in Ph‐positive ALL cell lines with those in each of 6 representative translocations among 87 BCP‐ALL cell lines. The p‐value in the Mann–Whitney analysis is indicated at the top when significant. Figure S2. Correlation between gene and cell surface expression levels of CD49f. Correlation between gene (horizontal axis) and cell surface expression (%; vertical axis) levels of CD49f in 27 BCP‐ALL cell lines, including 6 Ph‐positive ALL cell lines (red circles). The correlation coefficient is indicated at the top. Figure S3. CD29 and CD104 gene expression levels in Ph‐positive ALL samples. (A and B) Association of representative chromosomal abnormalities with CD29 gene expression (vertical axis) levels in childhood BCP‐ALL samples of the NOPHO (A) and St. Jude (B) cohorts. (C and D) Association of representative chromosomal abnormalities with CD104 gene expression (vertical axis) levels in childhood BCP‐ALL samples of the NOPHO (C) and St. Jude (D) cohorts. Figure S4. Laminin adhesion of Ph‐positive ALL cell lines. Images of three Ph‐positive ALL cell lines (KOPN30bi, KOPN55bi, KOPN66bi) attached to laminin‐uncoated (−) (upper panel) or laminin‐coated (+) (lower panel) plates after DAPI staining. Figure S5. Experimental workflow of blocking assay of laminin adhesion by specific antibodies. Figure S6. Laminin‐adhesion of Ph‐positive ALL cell lines through the CD49f‐CD29 heterodimer. Effects of anti‐CD49f (A and B), anti‐CD29 (C and D), and anti‐CD104 (E and F) blocking antibodies or isotype IgG (A, C, andE). Images of three Ph‐positive ALL cell lines attached to laminin‐uncoated (−) or laminin‐coated (+) plates after DAPI staining. (B, D, and F) Comparison of numbers of attached cell nuclei on laminin‐uncoated (−) or laminin‐coated (+) plates. The vertical axis indicates number of attached cell nuclei. The p‐value in the paired t‐test is indic [file CNR2-7-e2034-s001.zip › Figure S3D.jpg]

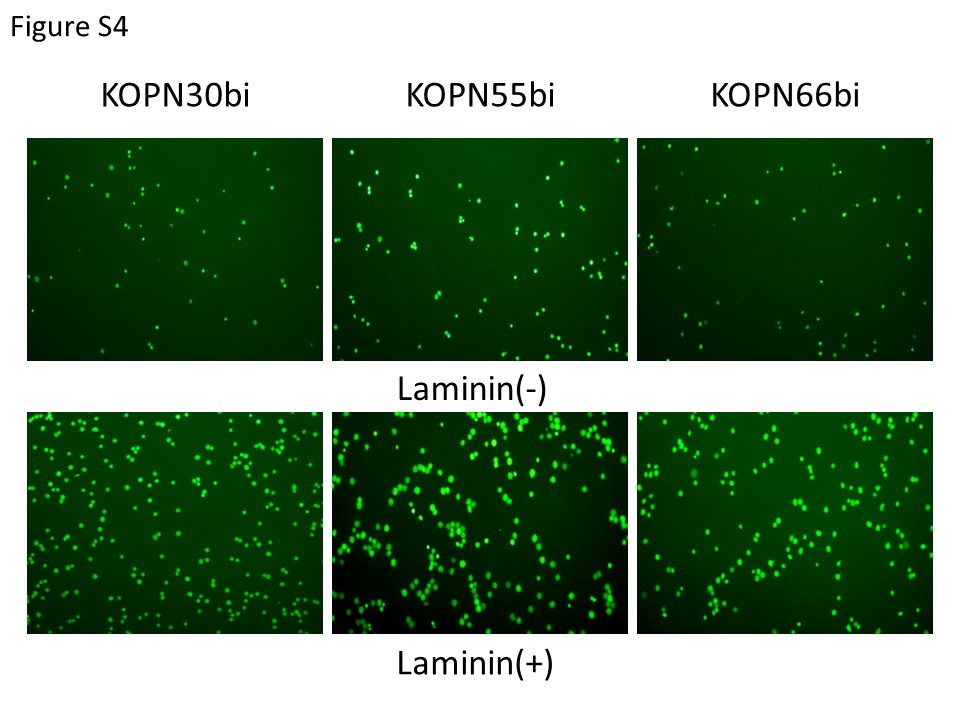

Supplement: Supplementary file 1 — Figure S1. High CD49f expression levels in Ph‐positive ALL. Comparison of cell surface CD49f expression (%; vertical axis) levels in Ph‐positive ALL cell lines with those in each of 6 representative translocations among 87 BCP‐ALL cell lines. The p‐value in the Mann–Whitney analysis is indicated at the top when significant. Figure S2. Correlation between gene and cell surface expression levels of CD49f. Correlation between gene (horizontal axis) and cell surface expression (%; vertical axis) levels of CD49f in 27 BCP‐ALL cell lines, including 6 Ph‐positive ALL cell lines (red circles). The correlation coefficient is indicated at the top. Figure S3. CD29 and CD104 gene expression levels in Ph‐positive ALL samples. (A and B) Association of representative chromosomal abnormalities with CD29 gene expression (vertical axis) levels in childhood BCP‐ALL samples of the NOPHO (A) and St. Jude (B) cohorts. (C and D) Association of representative chromosomal abnormalities with CD104 gene expression (vertical axis) levels in childhood BCP‐ALL samples of the NOPHO (C) and St. Jude (D) cohorts. Figure S4. Laminin adhesion of Ph‐positive ALL cell lines. Images of three Ph‐positive ALL cell lines (KOPN30bi, KOPN55bi, KOPN66bi) attached to laminin‐uncoated (−) (upper panel) or laminin‐coated (+) (lower panel) plates after DAPI staining. Figure S5. Experimental workflow of blocking assay of laminin adhesion by specific antibodies. Figure S6. Laminin‐adhesion of Ph‐positive ALL cell lines through the CD49f‐CD29 heterodimer. Effects of anti‐CD49f (A and B), anti‐CD29 (C and D), and anti‐CD104 (E and F) blocking antibodies or isotype IgG (A, C, andE). Images of three Ph‐positive ALL cell lines attached to laminin‐uncoated (−) or laminin‐coated (+) plates after DAPI staining. (B, D, and F) Comparison of numbers of attached cell nuclei on laminin‐uncoated (−) or laminin‐coated (+) plates. The vertical axis indicates number of attached cell nuclei. The p‐value in the paired t‐test is indic [file CNR2-7-e2034-s001.zip › Figure S4.jpg]

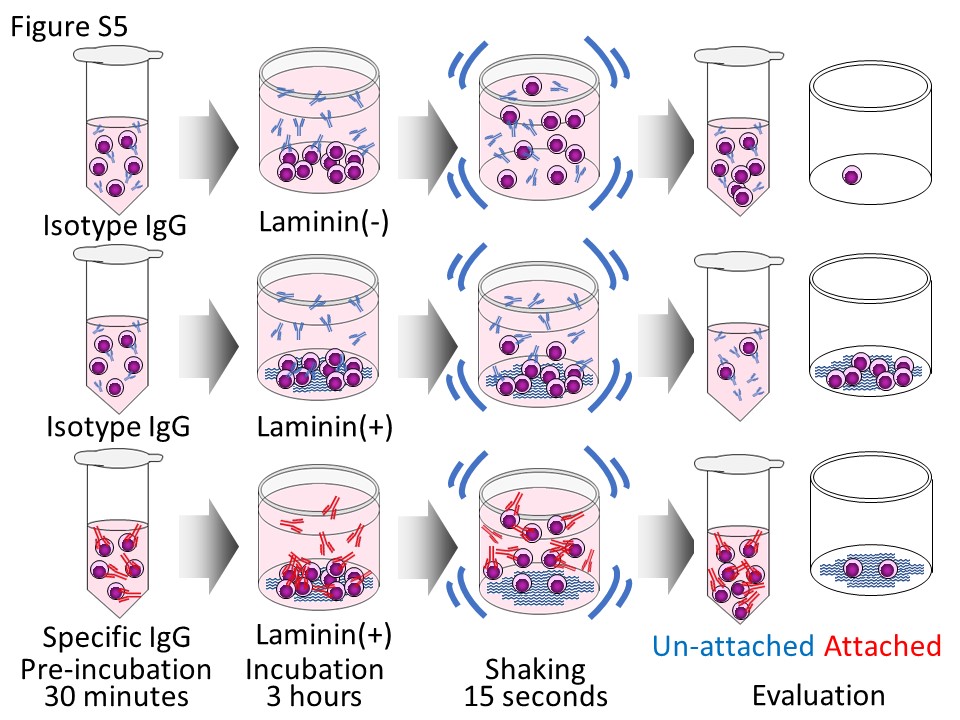

Supplement: Supplementary file 1 — Figure S1. High CD49f expression levels in Ph‐positive ALL. Comparison of cell surface CD49f expression (%; vertical axis) levels in Ph‐positive ALL cell lines with those in each of 6 representative translocations among 87 BCP‐ALL cell lines. The p‐value in the Mann–Whitney analysis is indicated at the top when significant. Figure S2. Correlation between gene and cell surface expression levels of CD49f. Correlation between gene (horizontal axis) and cell surface expression (%; vertical axis) levels of CD49f in 27 BCP‐ALL cell lines, including 6 Ph‐positive ALL cell lines (red circles). The correlation coefficient is indicated at the top. Figure S3. CD29 and CD104 gene expression levels in Ph‐positive ALL samples. (A and B) Association of representative chromosomal abnormalities with CD29 gene expression (vertical axis) levels in childhood BCP‐ALL samples of the NOPHO (A) and St. Jude (B) cohorts. (C and D) Association of representative chromosomal abnormalities with CD104 gene expression (vertical axis) levels in childhood BCP‐ALL samples of the NOPHO (C) and St. Jude (D) cohorts. Figure S4. Laminin adhesion of Ph‐positive ALL cell lines. Images of three Ph‐positive ALL cell lines (KOPN30bi, KOPN55bi, KOPN66bi) attached to laminin‐uncoated (−) (upper panel) or laminin‐coated (+) (lower panel) plates after DAPI staining. Figure S5. Experimental workflow of blocking assay of laminin adhesion by specific antibodies. Figure S6. Laminin‐adhesion of Ph‐positive ALL cell lines through the CD49f‐CD29 heterodimer. Effects of anti‐CD49f (A and B), anti‐CD29 (C and D), and anti‐CD104 (E and F) blocking antibodies or isotype IgG (A, C, andE). Images of three Ph‐positive ALL cell lines attached to laminin‐uncoated (−) or laminin‐coated (+) plates after DAPI staining. (B, D, and F) Comparison of numbers of attached cell nuclei on laminin‐uncoated (−) or laminin‐coated (+) plates. The vertical axis indicates number of attached cell nuclei. The p‐value in the paired t‐test is indic [file CNR2-7-e2034-s001.zip › Figure S5.jpg]

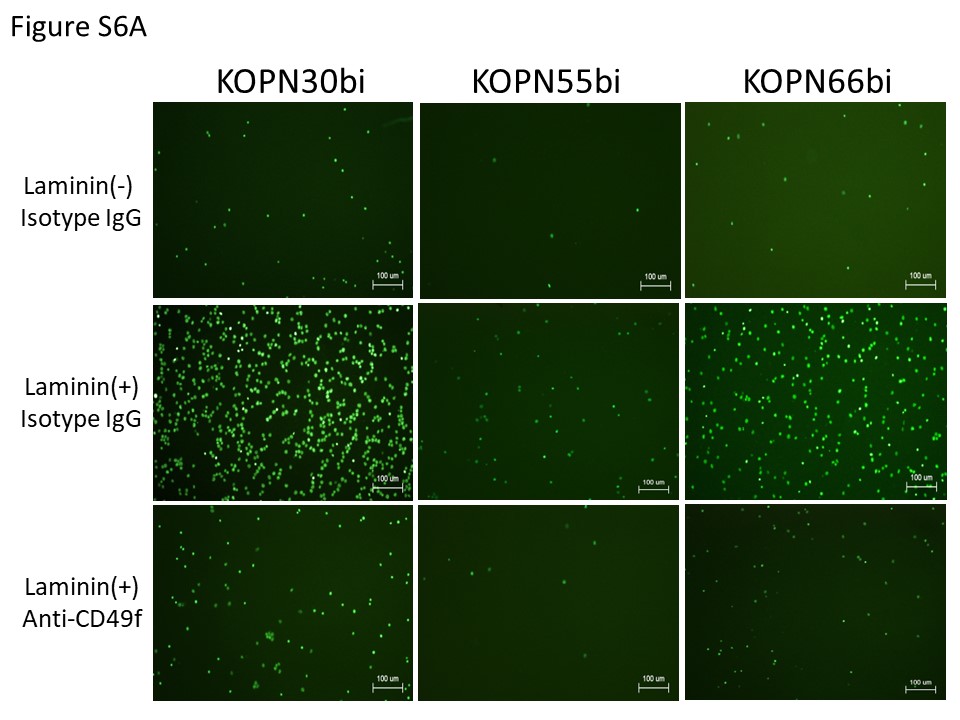

Supplement: Supplementary file 1 — Figure S1. High CD49f expression levels in Ph‐positive ALL. Comparison of cell surface CD49f expression (%; vertical axis) levels in Ph‐positive ALL cell lines with those in each of 6 representative translocations among 87 BCP‐ALL cell lines. The p‐value in the Mann–Whitney analysis is indicated at the top when significant. Figure S2. Correlation between gene and cell surface expression levels of CD49f. Correlation between gene (horizontal axis) and cell surface expression (%; vertical axis) levels of CD49f in 27 BCP‐ALL cell lines, including 6 Ph‐positive ALL cell lines (red circles). The correlation coefficient is indicated at the top. Figure S3. CD29 and CD104 gene expression levels in Ph‐positive ALL samples. (A and B) Association of representative chromosomal abnormalities with CD29 gene expression (vertical axis) levels in childhood BCP‐ALL samples of the NOPHO (A) and St. Jude (B) cohorts. (C and D) Association of representative chromosomal abnormalities with CD104 gene expression (vertical axis) levels in childhood BCP‐ALL samples of the NOPHO (C) and St. Jude (D) cohorts. Figure S4. Laminin adhesion of Ph‐positive ALL cell lines. Images of three Ph‐positive ALL cell lines (KOPN30bi, KOPN55bi, KOPN66bi) attached to laminin‐uncoated (−) (upper panel) or laminin‐coated (+) (lower panel) plates after DAPI staining. Figure S5. Experimental workflow of blocking assay of laminin adhesion by specific antibodies. Figure S6. Laminin‐adhesion of Ph‐positive ALL cell lines through the CD49f‐CD29 heterodimer. Effects of anti‐CD49f (A and B), anti‐CD29 (C and D), and anti‐CD104 (E and F) blocking antibodies or isotype IgG (A, C, andE). Images of three Ph‐positive ALL cell lines attached to laminin‐uncoated (−) or laminin‐coated (+) plates after DAPI staining. (B, D, and F) Comparison of numbers of attached cell nuclei on laminin‐uncoated (−) or laminin‐coated (+) plates. The vertical axis indicates number of attached cell nuclei. The p‐value in the paired t‐test is indic [file CNR2-7-e2034-s001.zip › Figure S6A.jpg]

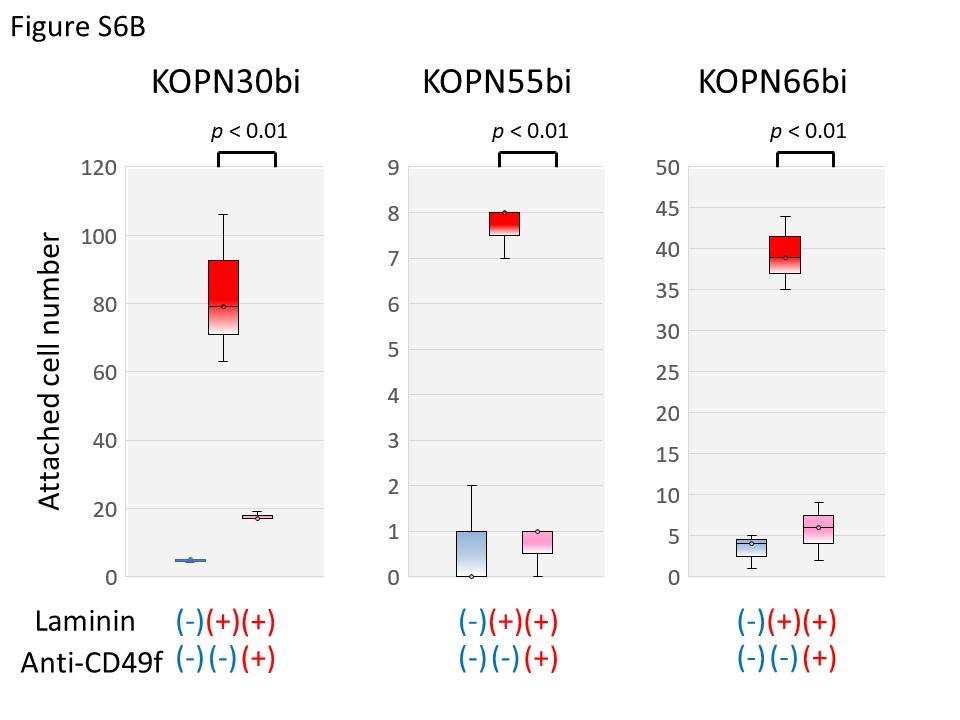

Supplement: Supplementary file 1 — Figure S1. High CD49f expression levels in Ph‐positive ALL. Comparison of cell surface CD49f expression (%; vertical axis) levels in Ph‐positive ALL cell lines with those in each of 6 representative translocations among 87 BCP‐ALL cell lines. The p‐value in the Mann–Whitney analysis is indicated at the top when significant. Figure S2. Correlation between gene and cell surface expression levels of CD49f. Correlation between gene (horizontal axis) and cell surface expression (%; vertical axis) levels of CD49f in 27 BCP‐ALL cell lines, including 6 Ph‐positive ALL cell lines (red circles). The correlation coefficient is indicated at the top. Figure S3. CD29 and CD104 gene expression levels in Ph‐positive ALL samples. (A and B) Association of representative chromosomal abnormalities with CD29 gene expression (vertical axis) levels in childhood BCP‐ALL samples of the NOPHO (A) and St. Jude (B) cohorts. (C and D) Association of representative chromosomal abnormalities with CD104 gene expression (vertical axis) levels in childhood BCP‐ALL samples of the NOPHO (C) and St. Jude (D) cohorts. Figure S4. Laminin adhesion of Ph‐positive ALL cell lines. Images of three Ph‐positive ALL cell lines (KOPN30bi, KOPN55bi, KOPN66bi) attached to laminin‐uncoated (−) (upper panel) or laminin‐coated (+) (lower panel) plates after DAPI staining. Figure S5. Experimental workflow of blocking assay of laminin adhesion by specific antibodies. Figure S6. Laminin‐adhesion of Ph‐positive ALL cell lines through the CD49f‐CD29 heterodimer. Effects of anti‐CD49f (A and B), anti‐CD29 (C and D), and anti‐CD104 (E and F) blocking antibodies or isotype IgG (A, C, andE). Images of three Ph‐positive ALL cell lines attached to laminin‐uncoated (−) or laminin‐coated (+) plates after DAPI staining. (B, D, and F) Comparison of numbers of attached cell nuclei on laminin‐uncoated (−) or laminin‐coated (+) plates. The vertical axis indicates number of attached cell nuclei. The p‐value in the paired t‐test is indic [file CNR2-7-e2034-s001.zip › Figure S6B.jpg]

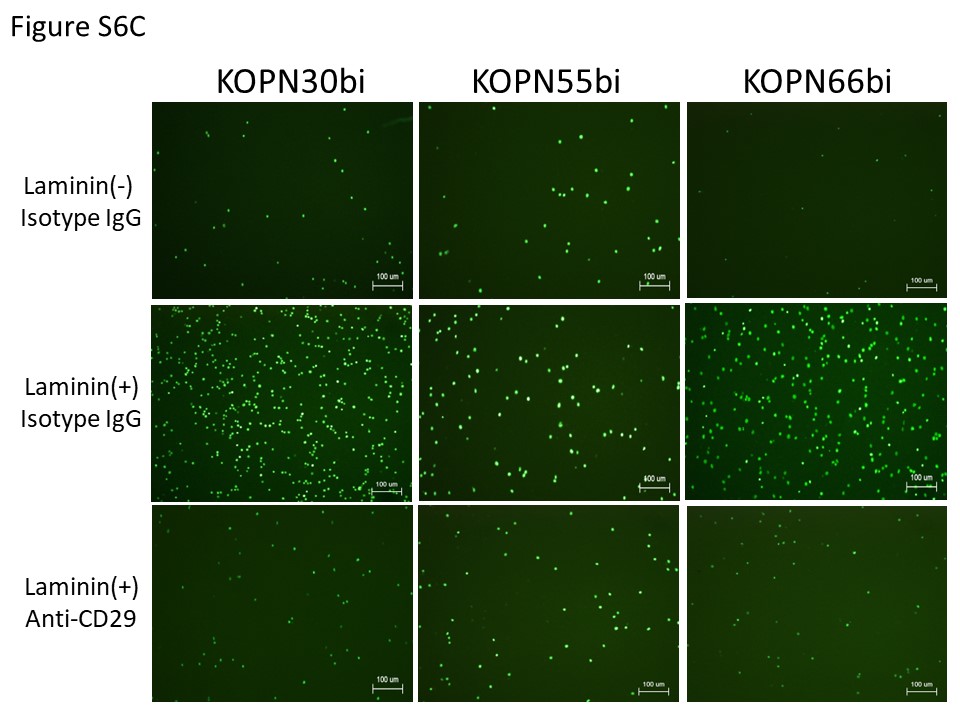

Supplement: Supplementary file 1 — Figure S1. High CD49f expression levels in Ph‐positive ALL. Comparison of cell surface CD49f expression (%; vertical axis) levels in Ph‐positive ALL cell lines with those in each of 6 representative translocations among 87 BCP‐ALL cell lines. The p‐value in the Mann–Whitney analysis is indicated at the top when significant. Figure S2. Correlation between gene and cell surface expression levels of CD49f. Correlation between gene (horizontal axis) and cell surface expression (%; vertical axis) levels of CD49f in 27 BCP‐ALL cell lines, including 6 Ph‐positive ALL cell lines (red circles). The correlation coefficient is indicated at the top. Figure S3. CD29 and CD104 gene expression levels in Ph‐positive ALL samples. (A and B) Association of representative chromosomal abnormalities with CD29 gene expression (vertical axis) levels in childhood BCP‐ALL samples of the NOPHO (A) and St. Jude (B) cohorts. (C and D) Association of representative chromosomal abnormalities with CD104 gene expression (vertical axis) levels in childhood BCP‐ALL samples of the NOPHO (C) and St. Jude (D) cohorts. Figure S4. Laminin adhesion of Ph‐positive ALL cell lines. Images of three Ph‐positive ALL cell lines (KOPN30bi, KOPN55bi, KOPN66bi) attached to laminin‐uncoated (−) (upper panel) or laminin‐coated (+) (lower panel) plates after DAPI staining. Figure S5. Experimental workflow of blocking assay of laminin adhesion by specific antibodies. Figure S6. Laminin‐adhesion of Ph‐positive ALL cell lines through the CD49f‐CD29 heterodimer. Effects of anti‐CD49f (A and B), anti‐CD29 (C and D), and anti‐CD104 (E and F) blocking antibodies or isotype IgG (A, C, andE). Images of three Ph‐positive ALL cell lines attached to laminin‐uncoated (−) or laminin‐coated (+) plates after DAPI staining. (B, D, and F) Comparison of numbers of attached cell nuclei on laminin‐uncoated (−) or laminin‐coated (+) plates. The vertical axis indicates number of attached cell nuclei. The p‐value in the paired t‐test is indic [file CNR2-7-e2034-s001.zip › Figure S6C.jpg]

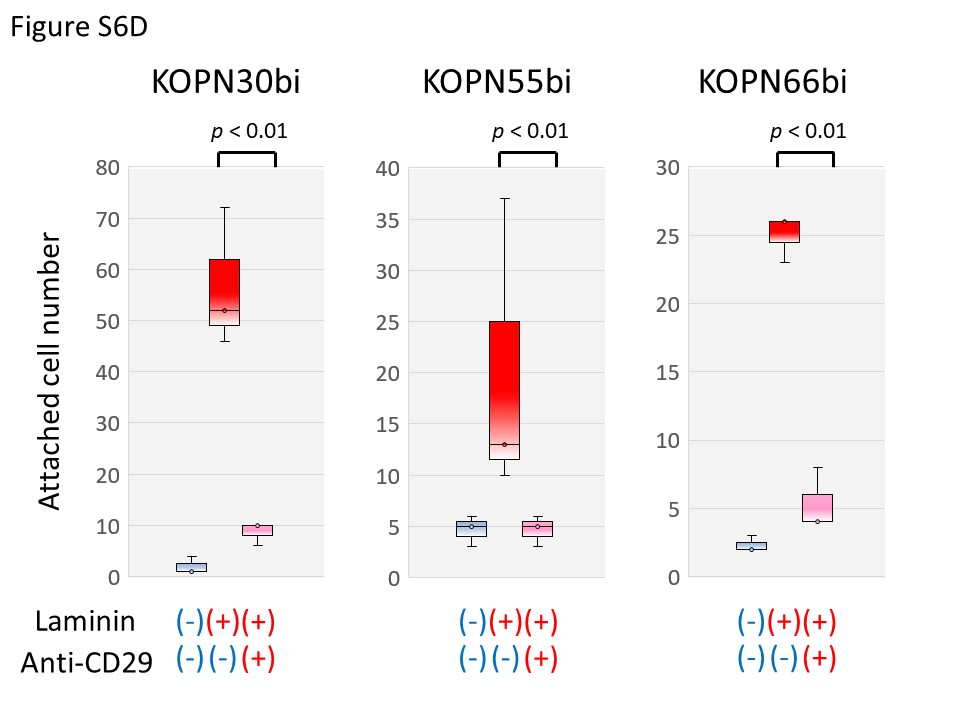

Supplement: Supplementary file 1 — Figure S1. High CD49f expression levels in Ph‐positive ALL. Comparison of cell surface CD49f expression (%; vertical axis) levels in Ph‐positive ALL cell lines with those in each of 6 representative translocations among 87 BCP‐ALL cell lines. The p‐value in the Mann–Whitney analysis is indicated at the top when significant. Figure S2. Correlation between gene and cell surface expression levels of CD49f. Correlation between gene (horizontal axis) and cell surface expression (%; vertical axis) levels of CD49f in 27 BCP‐ALL cell lines, including 6 Ph‐positive ALL cell lines (red circles). The correlation coefficient is indicated at the top. Figure S3. CD29 and CD104 gene expression levels in Ph‐positive ALL samples. (A and B) Association of representative chromosomal abnormalities with CD29 gene expression (vertical axis) levels in childhood BCP‐ALL samples of the NOPHO (A) and St. Jude (B) cohorts. (C and D) Association of representative chromosomal abnormalities with CD104 gene expression (vertical axis) levels in childhood BCP‐ALL samples of the NOPHO (C) and St. Jude (D) cohorts. Figure S4. Laminin adhesion of Ph‐positive ALL cell lines. Images of three Ph‐positive ALL cell lines (KOPN30bi, KOPN55bi, KOPN66bi) attached to laminin‐uncoated (−) (upper panel) or laminin‐coated (+) (lower panel) plates after DAPI staining. Figure S5. Experimental workflow of blocking assay of laminin adhesion by specific antibodies. Figure S6. Laminin‐adhesion of Ph‐positive ALL cell lines through the CD49f‐CD29 heterodimer. Effects of anti‐CD49f (A and B), anti‐CD29 (C and D), and anti‐CD104 (E and F) blocking antibodies or isotype IgG (A, C, andE). Images of three Ph‐positive ALL cell lines attached to laminin‐uncoated (−) or laminin‐coated (+) plates after DAPI staining. (B, D, and F) Comparison of numbers of attached cell nuclei on laminin‐uncoated (−) or laminin‐coated (+) plates. The vertical axis indicates number of attached cell nuclei. The p‐value in the paired t‐test is indic [file CNR2-7-e2034-s001.zip › Figure S6D.jpg]

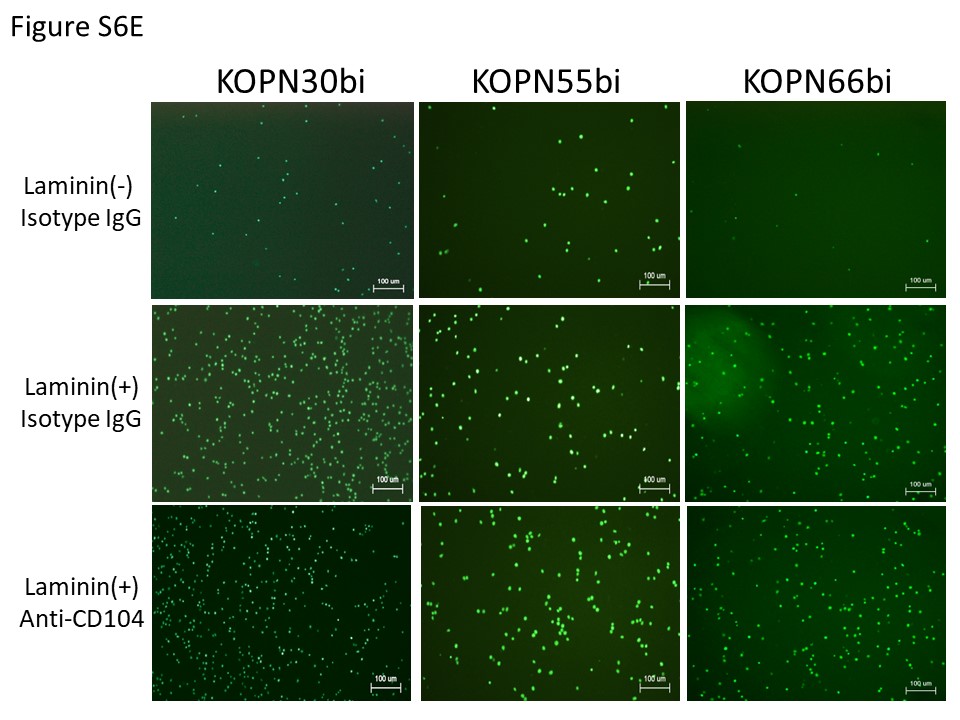

Supplement: Supplementary file 1 — Figure S1. High CD49f expression levels in Ph‐positive ALL. Comparison of cell surface CD49f expression (%; vertical axis) levels in Ph‐positive ALL cell lines with those in each of 6 representative translocations among 87 BCP‐ALL cell lines. The p‐value in the Mann–Whitney analysis is indicated at the top when significant. Figure S2. Correlation between gene and cell surface expression levels of CD49f. Correlation between gene (horizontal axis) and cell surface expression (%; vertical axis) levels of CD49f in 27 BCP‐ALL cell lines, including 6 Ph‐positive ALL cell lines (red circles). The correlation coefficient is indicated at the top. Figure S3. CD29 and CD104 gene expression levels in Ph‐positive ALL samples. (A and B) Association of representative chromosomal abnormalities with CD29 gene expression (vertical axis) levels in childhood BCP‐ALL samples of the NOPHO (A) and St. Jude (B) cohorts. (C and D) Association of representative chromosomal abnormalities with CD104 gene expression (vertical axis) levels in childhood BCP‐ALL samples of the NOPHO (C) and St. Jude (D) cohorts. Figure S4. Laminin adhesion of Ph‐positive ALL cell lines. Images of three Ph‐positive ALL cell lines (KOPN30bi, KOPN55bi, KOPN66bi) attached to laminin‐uncoated (−) (upper panel) or laminin‐coated (+) (lower panel) plates after DAPI staining. Figure S5. Experimental workflow of blocking assay of laminin adhesion by specific antibodies. Figure S6. Laminin‐adhesion of Ph‐positive ALL cell lines through the CD49f‐CD29 heterodimer. Effects of anti‐CD49f (A and B), anti‐CD29 (C and D), and anti‐CD104 (E and F) blocking antibodies or isotype IgG (A, C, andE). Images of three Ph‐positive ALL cell lines attached to laminin‐uncoated (−) or laminin‐coated (+) plates after DAPI staining. (B, D, and F) Comparison of numbers of attached cell nuclei on laminin‐uncoated (−) or laminin‐coated (+) plates. The vertical axis indicates number of attached cell nuclei. The p‐value in the paired t‐test is indic [file CNR2-7-e2034-s001.zip › Figure S6E.jpg]

Figure S6F

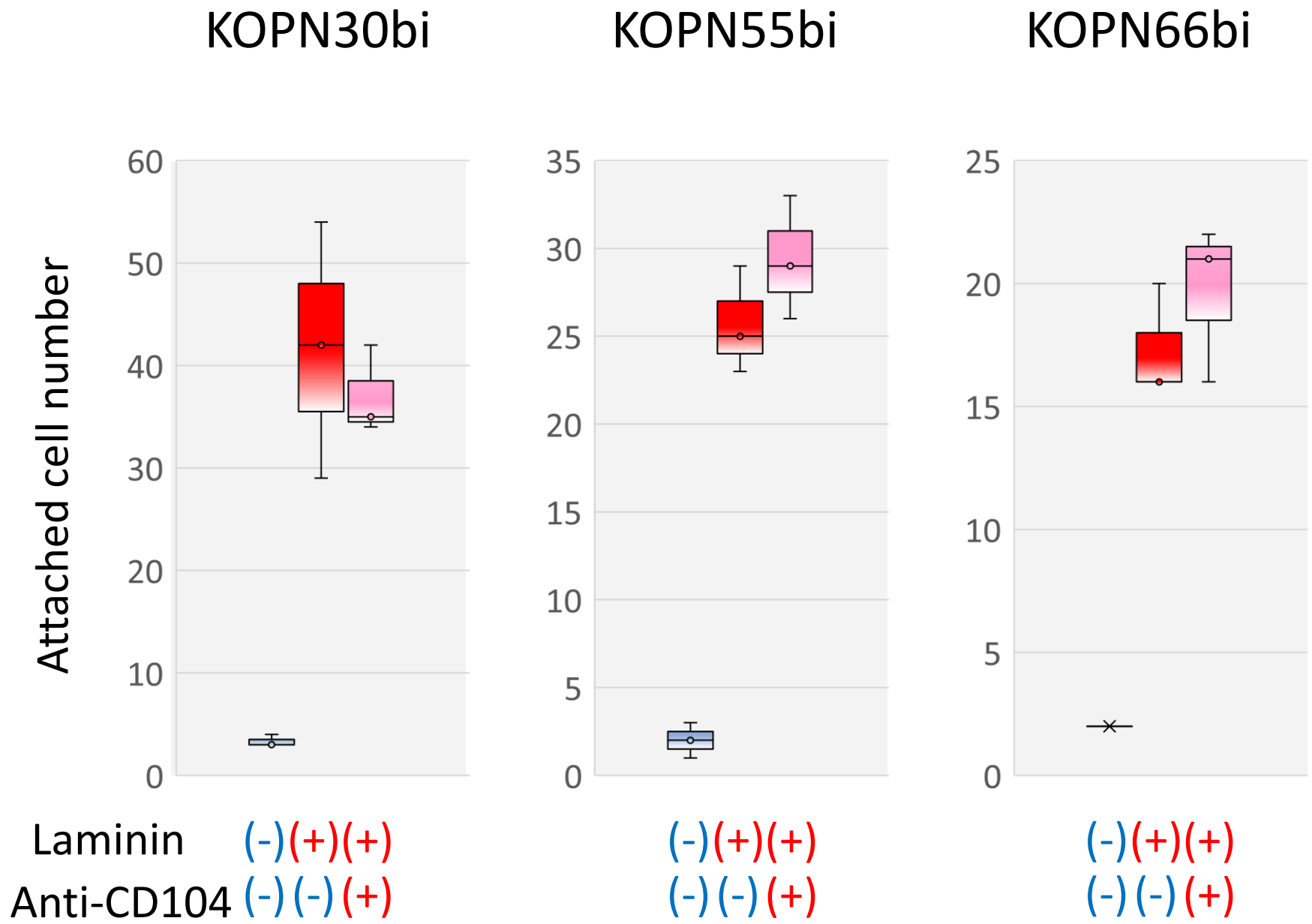

Supplement: Supplementary file 1 — Figure S1. High CD49f expression levels in Ph‐positive ALL. Comparison of cell surface CD49f expression (%; vertical axis) levels in Ph‐positive ALL cell lines with those in each of 6 representative translocations among 87 BCP‐ALL cell lines. The p‐value in the Mann–Whitney analysis is indicated at the top when significant. Figure S2. Correlation between gene and cell surface expression levels of CD49f. Correlation between gene (horizontal axis) and cell surface expression (%; vertical axis) levels of CD49f in 27 BCP‐ALL cell lines, including 6 Ph‐positive ALL cell lines (red circles). The correlation coefficient is indicated at the top. Figure S3. CD29 and CD104 gene expression levels in Ph‐positive ALL samples. (A and B) Association of representative chromosomal abnormalities with CD29 gene expression (vertical axis) levels in childhood BCP‐ALL samples of the NOPHO (A) and St. Jude (B) cohorts. (C and D) Association of representative chromosomal abnormalities with CD104 gene expression (vertical axis) levels in childhood BCP‐ALL samples of the NOPHO (C) and St. Jude (D) cohorts. Figure S4. Laminin adhesion of Ph‐positive ALL cell lines. Images of three Ph‐positive ALL cell lines (KOPN30bi, KOPN55bi, KOPN66bi) attached to laminin‐uncoated (−) (upper panel) or laminin‐coated (+) (lower panel) plates after DAPI staining. Figure S5. Experimental workflow of blocking assay of laminin adhesion by specific antibodies. Figure S6. Laminin‐adhesion of Ph‐positive ALL cell lines through the CD49f‐CD29 heterodimer. Effects of anti‐CD49f (A and B), anti‐CD29 (C and D), and anti‐CD104 (E and F) blocking antibodies or isotype IgG (A, C, andE). Images of three Ph‐positive ALL cell lines attached to laminin‐uncoated (−) or laminin‐coated (+) plates after DAPI staining. (B, D, and F) Comparison of numbers of attached cell nuclei on laminin‐uncoated (−) or laminin‐coated (+) plates. The vertical axis indicates number of attached cell nuclei. The p‐value in the paired t‐test is indic [file CNR2-7-e2034-s001.zip › Figure S6F revision.pdf]

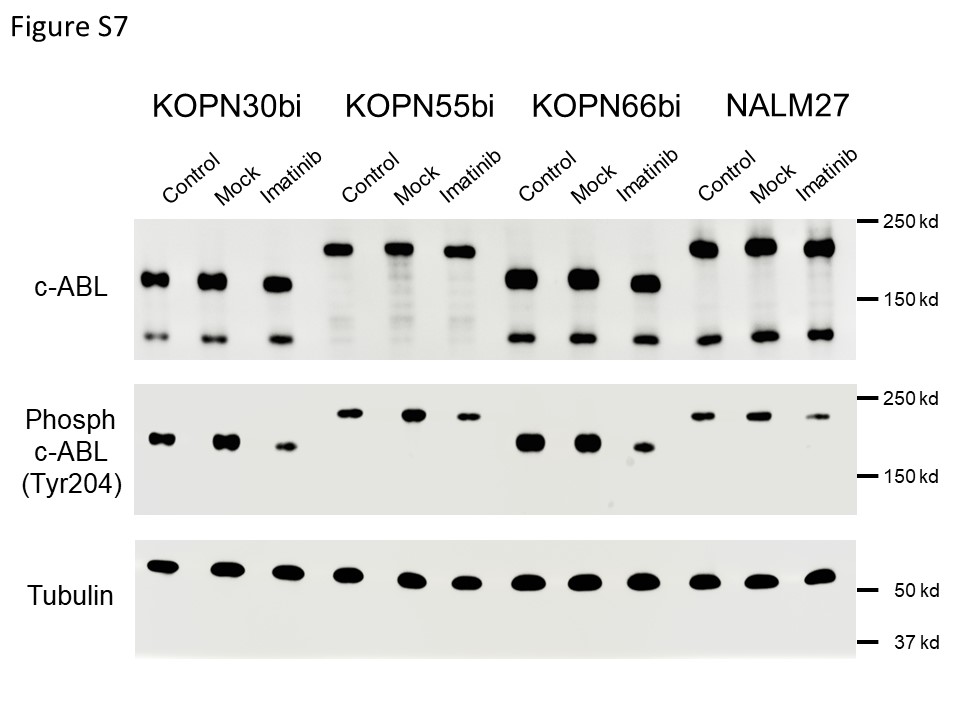

Supplement: Supplementary file 1 — Figure S1. High CD49f expression levels in Ph‐positive ALL. Comparison of cell surface CD49f expression (%; vertical axis) levels in Ph‐positive ALL cell lines with those in each of 6 representative translocations among 87 BCP‐ALL cell lines. The p‐value in the Mann–Whitney analysis is indicated at the top when significant. Figure S2. Correlation between gene and cell surface expression levels of CD49f. Correlation between gene (horizontal axis) and cell surface expression (%; vertical axis) levels of CD49f in 27 BCP‐ALL cell lines, including 6 Ph‐positive ALL cell lines (red circles). The correlation coefficient is indicated at the top. Figure S3. CD29 and CD104 gene expression levels in Ph‐positive ALL samples. (A and B) Association of representative chromosomal abnormalities with CD29 gene expression (vertical axis) levels in childhood BCP‐ALL samples of the NOPHO (A) and St. Jude (B) cohorts. (C and D) Association of representative chromosomal abnormalities with CD104 gene expression (vertical axis) levels in childhood BCP‐ALL samples of the NOPHO (C) and St. Jude (D) cohorts. Figure S4. Laminin adhesion of Ph‐positive ALL cell lines. Images of three Ph‐positive ALL cell lines (KOPN30bi, KOPN55bi, KOPN66bi) attached to laminin‐uncoated (−) (upper panel) or laminin‐coated (+) (lower panel) plates after DAPI staining. Figure S5. Experimental workflow of blocking assay of laminin adhesion by specific antibodies. Figure S6. Laminin‐adhesion of Ph‐positive ALL cell lines through the CD49f‐CD29 heterodimer. Effects of anti‐CD49f (A and B), anti‐CD29 (C and D), and anti‐CD104 (E and F) blocking antibodies or isotype IgG (A, C, andE). Images of three Ph‐positive ALL cell lines attached to laminin‐uncoated (−) or laminin‐coated (+) plates after DAPI staining. (B, D, and F) Comparison of numbers of attached cell nuclei on laminin‐uncoated (−) or laminin‐coated (+) plates. The vertical axis indicates number of attached cell nuclei. The p‐value in the paired t‐test is indic [file CNR2-7-e2034-s001.zip › Figure S7.jpg]

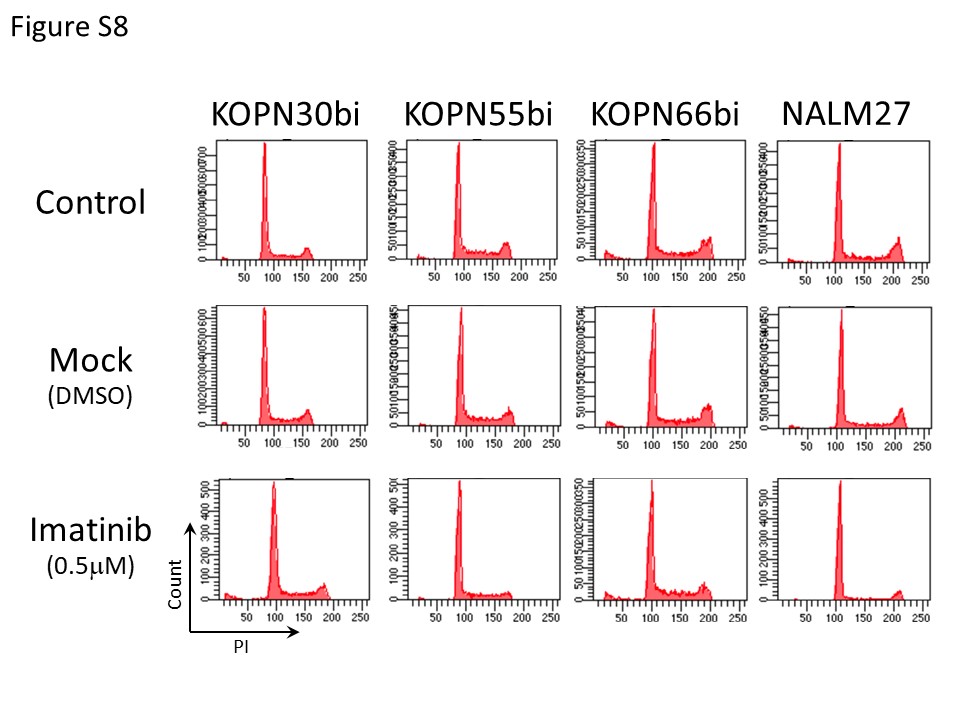

Supplement: Supplementary file 1 — Figure S1. High CD49f expression levels in Ph‐positive ALL. Comparison of cell surface CD49f expression (%; vertical axis) levels in Ph‐positive ALL cell lines with those in each of 6 representative translocations among 87 BCP‐ALL cell lines. The p‐value in the Mann–Whitney analysis is indicated at the top when significant. Figure S2. Correlation between gene and cell surface expression levels of CD49f. Correlation between gene (horizontal axis) and cell surface expression (%; vertical axis) levels of CD49f in 27 BCP‐ALL cell lines, including 6 Ph‐positive ALL cell lines (red circles). The correlation coefficient is indicated at the top. Figure S3. CD29 and CD104 gene expression levels in Ph‐positive ALL samples. (A and B) Association of representative chromosomal abnormalities with CD29 gene expression (vertical axis) levels in childhood BCP‐ALL samples of the NOPHO (A) and St. Jude (B) cohorts. (C and D) Association of representative chromosomal abnormalities with CD104 gene expression (vertical axis) levels in childhood BCP‐ALL samples of the NOPHO (C) and St. Jude (D) cohorts. Figure S4. Laminin adhesion of Ph‐positive ALL cell lines. Images of three Ph‐positive ALL cell lines (KOPN30bi, KOPN55bi, KOPN66bi) attached to laminin‐uncoated (−) (upper panel) or laminin‐coated (+) (lower panel) plates after DAPI staining. Figure S5. Experimental workflow of blocking assay of laminin adhesion by specific antibodies. Figure S6. Laminin‐adhesion of Ph‐positive ALL cell lines through the CD49f‐CD29 heterodimer. Effects of anti‐CD49f (A and B), anti‐CD29 (C and D), and anti‐CD104 (E and F) blocking antibodies or isotype IgG (A, C, andE). Images of three Ph‐positive ALL cell lines attached to laminin‐uncoated (−) or laminin‐coated (+) plates after DAPI staining. (B, D, and F) Comparison of numbers of attached cell nuclei on laminin‐uncoated (−) or laminin‐coated (+) plates. The vertical axis indicates number of attached cell nuclei. The p‐value in the paired t‐test is indic [file CNR2-7-e2034-s001.zip › Figure S8.jpg]

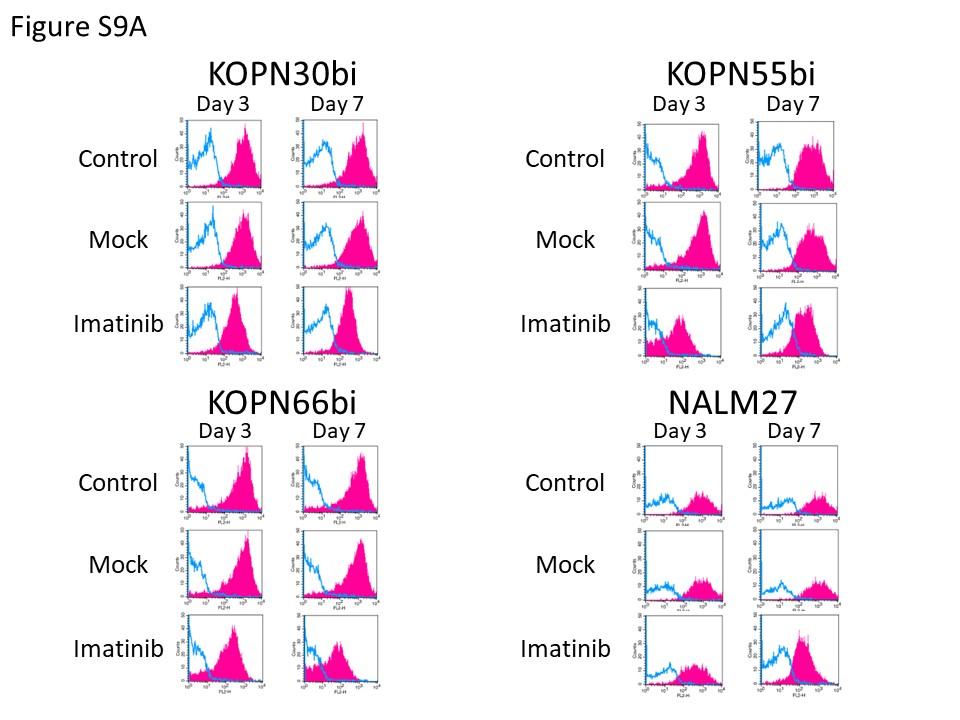

Supplement: Supplementary file 1 — Figure S1. High CD49f expression levels in Ph‐positive ALL. Comparison of cell surface CD49f expression (%; vertical axis) levels in Ph‐positive ALL cell lines with those in each of 6 representative translocations among 87 BCP‐ALL cell lines. The p‐value in the Mann–Whitney analysis is indicated at the top when significant. Figure S2. Correlation between gene and cell surface expression levels of CD49f. Correlation between gene (horizontal axis) and cell surface expression (%; vertical axis) levels of CD49f in 27 BCP‐ALL cell lines, including 6 Ph‐positive ALL cell lines (red circles). The correlation coefficient is indicated at the top. Figure S3. CD29 and CD104 gene expression levels in Ph‐positive ALL samples. (A and B) Association of representative chromosomal abnormalities with CD29 gene expression (vertical axis) levels in childhood BCP‐ALL samples of the NOPHO (A) and St. Jude (B) cohorts. (C and D) Association of representative chromosomal abnormalities with CD104 gene expression (vertical axis) levels in childhood BCP‐ALL samples of the NOPHO (C) and St. Jude (D) cohorts. Figure S4. Laminin adhesion of Ph‐positive ALL cell lines. Images of three Ph‐positive ALL cell lines (KOPN30bi, KOPN55bi, KOPN66bi) attached to laminin‐uncoated (−) (upper panel) or laminin‐coated (+) (lower panel) plates after DAPI staining. Figure S5. Experimental workflow of blocking assay of laminin adhesion by specific antibodies. Figure S6. Laminin‐adhesion of Ph‐positive ALL cell lines through the CD49f‐CD29 heterodimer. Effects of anti‐CD49f (A and B), anti‐CD29 (C and D), and anti‐CD104 (E and F) blocking antibodies or isotype IgG (A, C, andE). Images of three Ph‐positive ALL cell lines attached to laminin‐uncoated (−) or laminin‐coated (+) plates after DAPI staining. (B, D, and F) Comparison of numbers of attached cell nuclei on laminin‐uncoated (−) or laminin‐coated (+) plates. The vertical axis indicates number of attached cell nuclei. The p‐value in the paired t‐test is indic [file CNR2-7-e2034-s001.zip › Figure S9A.jpg]

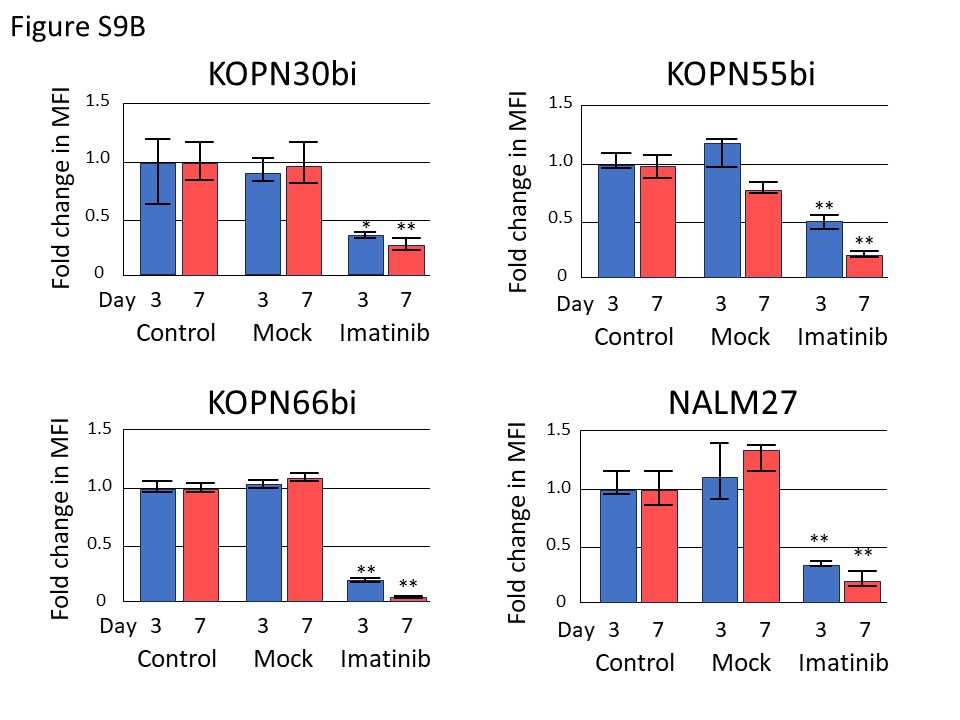

Supplement: Supplementary file 1 — Figure S1. High CD49f expression levels in Ph‐positive ALL. Comparison of cell surface CD49f expression (%; vertical axis) levels in Ph‐positive ALL cell lines with those in each of 6 representative translocations among 87 BCP‐ALL cell lines. The p‐value in the Mann–Whitney analysis is indicated at the top when significant. Figure S2. Correlation between gene and cell surface expression levels of CD49f. Correlation between gene (horizontal axis) and cell surface expression (%; vertical axis) levels of CD49f in 27 BCP‐ALL cell lines, including 6 Ph‐positive ALL cell lines (red circles). The correlation coefficient is indicated at the top. Figure S3. CD29 and CD104 gene expression levels in Ph‐positive ALL samples. (A and B) Association of representative chromosomal abnormalities with CD29 gene expression (vertical axis) levels in childhood BCP‐ALL samples of the NOPHO (A) and St. Jude (B) cohorts. (C and D) Association of representative chromosomal abnormalities with CD104 gene expression (vertical axis) levels in childhood BCP‐ALL samples of the NOPHO (C) and St. Jude (D) cohorts. Figure S4. Laminin adhesion of Ph‐positive ALL cell lines. Images of three Ph‐positive ALL cell lines (KOPN30bi, KOPN55bi, KOPN66bi) attached to laminin‐uncoated (−) (upper panel) or laminin‐coated (+) (lower panel) plates after DAPI staining. Figure S5. Experimental workflow of blocking assay of laminin adhesion by specific antibodies. Figure S6. Laminin‐adhesion of Ph‐positive ALL cell lines through the CD49f‐CD29 heterodimer. Effects of anti‐CD49f (A and B), anti‐CD29 (C and D), and anti‐CD104 (E and F) blocking antibodies or isotype IgG (A, C, andE). Images of three Ph‐positive ALL cell lines attached to laminin‐uncoated (−) or laminin‐coated (+) plates after DAPI staining. (B, D, and F) Comparison of numbers of attached cell nuclei on laminin‐uncoated (−) or laminin‐coated (+) plates. The vertical axis indicates number of attached cell nuclei. The p‐value in the paired t‐test is indic [file CNR2-7-e2034-s001.zip › Figure S9B.jpg]
